# Advancing the Potential of *Ostericum palustre* (Besser) Besser (Synonym *Angelica pancicii* Vandas ex. Velen.) of Bulgarian Origin as a Source of Bioactive Compounds: Metabolite Profiling and Pharmacological Activity

**DOI:** 10.3390/plants15081172

**Published:** 2026-04-10

**Authors:** Reneta Gevrenova, Gokhan Zengin, Kouadio Ibrahime Sinan, Inci Kurt-Celep, Alexandra Stefanova, Dimitrina Zheleva-Dimitrova

**Affiliations:** 1 Department of Pharmacognosy, Faculty of Pharmacy, Medical University-Sofia, 1000 Sofia, Bulgaria; 2 Physiology and Biochemistry Research Laboratory, Department of Biology, Science Faculty, Selcuk University, Konya 42130, Turkey; gokhanzengin@selcuk.edu.tr (G.Z.); sinankouadio@gmail.com (K.I.S.); 3 Department of Biochemistry-Genetics, Faculty of Biological Sciences, Educational and Research Unit of Genetic, University of Peleforo Gon Coulibaly (UPGC), Korhogo BP1328, Côte d’Ivoire; 4 Department of Pharmaceutical Biotechnology, Faculty of Pharmacy, İstanbul Okan University, Tuzla, İstanbul 34940, Turkey; inci.celep@okan.edu.tr; 5 Department of Pharmacology, Pharmacotherapy, and Toxicology, Faculty of Pharmacy, Medical University-Sofia, 1000 Sofia, Bulgaria; astefanova22@gmail.com

**Keywords:** *Angelica pancicii* Vandas ex. Velen., LC-MS profiling, coumarins, antioxidant activity, enzyme inhibitory activity

## Abstract

*Ostericum palustre* (Besser) Besser (synonym *Angelica pancicii* Vandas ex. Velen.) is a Eurasian species from the Apiaceae family, previously related to the Balkan endemic species *A. pancicii*. The study aims to provide a thorough profiling of methanol-aqueous extracts from *O. palustre* leaves, roots, and inflorescences integrated with an evaluation of antioxidant potential and enzyme inhibitory activity towards some therapeutic targets. For the first time, a series of simple coumarins and furanocoumarins alongside phenolic and acylquinic acids, and flavonoids were annotated/dereplicated in the *O. palustre* of Bulgarian origin by liquid chromatography coupled with quadrupole—Orbitrap high resolution mass spectrometry acquisition platform. According to the discriminant analysis (sPLS-DA) of the biological potential, radical scavenging activity (47.9 mg TE/g in DPPH and 61.8 mg TE/g in ABTS), reducing power (102.2 mg TE/g in CUPRAC and 57.4 mg TE/g in FRAP), and metal-chelating capacity (20.1 mg EDTAE/g) accounted mainly for the stronger antioxidant activity of inflorescences extract than roots and leaves. Root extracts exhibited anti-collagenase, anti-elastase, and anti-hyaluronidase effects with lower IC_50_ values (IC_50_ 37.22, 42.47 and 32.09 μg/mL, respectively). Pearson relationship analysis revealed potent antioxidants including furanocoumarins (oxypeucedanin hydrate, xanthotoxol/bergaptol, byakangelicin/isobyakangelicin, ostruthol) and phenolic acids, while a series of angelols alongside feruloylquinic and dicaffeoylquinic acids, and flavonol glycosides hold significance for the neuroprotective activity of the leaves extract. The enzyme inhibitory activity of the root extracts towards collagenase, elastase and hyaluronidase, related to the anti-aging activity, was ascribed to simple hydroxylated/methoxylated coumarins. The study suggests the potential health benefits of *O. palustre* extracts as antioxidant, anti-aging, and neuroprotective agents.

## 1. Introduction

*Ostericum palustre* (Besser) Besser (synonym *Angelica pancicii* Vandas ex. Velen.) is a perennial plant from the Apiaceae family. It is a Eurasian species distributed in the temperate zones of Middle and Southeastern Europe, and Western and Middle Asia [1]. The Kew database recognizes *O. palustre* as a type species and *A. pancicii* as a synonym. The Balkan population was previously considered as Balkan endemite *A. pancicii* in Bulgaria, Serbia, North Macedonia, Albania and Romania [2]. However, significant advances have been made in molecular phylogeny to evaluate the taxonomy of the genus *Angelica* L. As highlighted in recent examinations, *Angelica* is consistent with *Ostericum* in fruit and flower morphology but distantly linked to *Ostericum* in molecular phylogenetic studies [3,4]. Accumulating evidence based on DNA sequencing revealed significant differences between two genera, strongly supporting *O. palustre* type species. Despite the revised taxonomic position of the species, the literature survey of its phytochemistry showed that it was referred to as *A. pancicii* in the published data. Essential oils (EO) from *O. palustre*/*A. pancicii* aerial parts, fruits, leaves, and roots have been thoroughly analyzed [5,6,7]. EO accounted for 1.29, 0.07 and 0.13% from the fruits, leaves and roots, respectively [7]. More than 200 compounds have been reported in the EOs. Significant differences have been found between aerial parts EO of Serbian and Macedonian origin [5,6]. The former was dominated by monoterpenoids (92.8% and 97.7% in GC-MS and HS/GC-MS analyses, respectively), while the latter was characterized by a high quantity of oxygenated sesquiterpenoids (34.96%) and sesquiterpene hydrocarbons (21.88%) (GC-FID/GC-MS). On the other hand, EO from the *O. palustre*/*A. pancicii* fruits with Bulgarian provenance possessed monoterpene hydrocarbons (84.3%); leaf and root EOs were rich in sesquiterpenoids (64.0 and 71.7%, respectively) [7]. (+)-β-Phellandrene was the predominant monoterpene being present up to 60.1% in the aerial parts EO and 69.1% in the fruits EO [6,7]. Other EO constituents included: α-pinene, α-phellandrene, δ-3-carene (aerial parts), germacrene D, δ-cadinene and caryophyllene oxide (leaves), and elemol, kessane, and γ-eudesmol (roots).

Overall, numerous simple coumarins and furanocoumarins alongside their glycosides, furanocoumarin ethers, pyranocoumarins and chromones have been tentatively identified in *O. palustre*/*A. pancicii* aerial parts and roots by HPLC-DAD/ESI-TOF-MS [6]. Oxypeucedanin, oxypeucedanin hydrate and imperatorin have been isolated from *O. palustre*/*A. pancicii* fruits and roots; angeloylpangelin and umbelliprenin have been established in the fruits, while saxalin, ostrutol and 5′-acetylcnidimol A—in the roots [6,7]. There are scarce data on the use of *O. palustre*/*A. pancicii* roots as a medicinal plant with anti-hypertensive activity in Bulgarian traditional medicine [7]. Mileski et al. (2017) particularly emphasized aerial parts ethanol extracts for their antioxidant activity [6]. The authors have found strong to moderate antibacterial effects of the methanol and ethanol root extracts against Gram (+) bacteria along with anti-boifilm activity, comparable with the positive control streptomycin [6]. The leaves’ hexan extract actively scavenged free radicals (DPPH), while root extracts, rich in oxypeucedanin, inhibited α-amylase. Fruit and leaf EOs exerted significant inhibitory effects towards acetylcholinesterase [7].

It is noteworthy that the aforementioned studies were mainly focused on the constituents of *O. palustre*/*A. pancicii* EO and apolar extracts, while there is no in-depth metabolite profiling of the species by means of liquid chromatography—Orbitrap—high resolution mass spectrometry (LC-HRMS).

Owing to the fact that the center of the biodiversity of the *Ostericum* genus is Chaina (with 11 species out of 13 in the world), *O. citriodorum* (Hance) R.H.Shan and C.Q.Yuan, *O. grosseserratum* (Maxim.) Kitag. and *O. sieboldii* (Miq.) Nakai are valuable medicinal plants used the traditional Chinese and Korean Medicine [8]. The advances in the traditional uses and pharmacology of *O. grosseserratum* have recently been summarized [9]. Current pharmacological investigations of the *O. grosseserratum* roots demonstrated anti-tumor, anti-inflammatory, and acaricidal activity alongside vasorelaxant, anti-obesity and anti-diabetic effects [9,10]. Among the marker compounds sesquiterpene bisabolangelone together with the furanocoumarins oxypeucedanin, imperatorin, isoimperatorin, peucedanin and peucedanin hydrate have been found [11,12]. The aforementioned studies provide references for the further research of the unexplored *Ostericum* taxa. It is worth noting that *Angelica* herbal drugs are drawing increased interest from a phytopharmacological point of view and much of the studies have been centered on the most important species renowned for their use in Chinese and Korean traditional medicine (*A. dahurica*, *A. sinensis*, *A. archangelica*) [13,14]. A variety of coumarins, flavonoids, sterols, benzofurans, polyacetylenes and polysaccharides hold significance for the observed health-promoting effects of both *Angelica* and *Ostericum* taxa.

Taking these studies together, we undertook a thorough profiling of methanol-aqueous extracts from the leaves, roots, and inflorescences of *O. palustre* of Bulgarian origin by means of LC-Orbitrap-HRMS. This research was integrated with an evaluation of antioxidant potential through different mechanisms and enzyme inhibitory activity towards some chief therapeutic targets such as cholinesterases, tyrosinase, α-amylase, α-glucosidase, collagenase, elastase, hyaluronidase, and data was subjected to discriminant analysis.

## 2. Results and Discussion

### 2.1. UHPLC-HRMS Profiling

**Figure 1:**
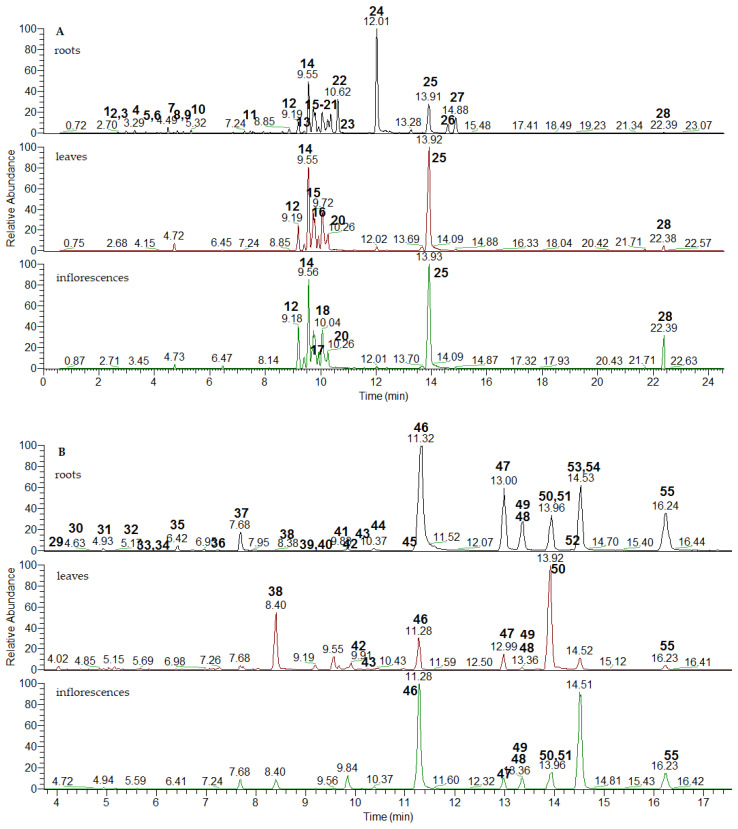
Extracted ion chromatograms of simple coumarins (**A**) and furanocumarins (**B**) proceed with mass tolerance of 5 ppm (for the *m*/*z* of [M + H]^+^ and mass ranges, see Supplementary data; for the peaks numbering see Table 1).

Herein, 28 simple coumarins, 26 furanocoumarins, 18 acylquinic acids, 19 flavonoid aglycones and glycosides, and 20 phenolic acids and their glycosides were reported in *O. palustre* (Table 1, Figure 1, Figures S1 and S2). To the best of our knowledge, 14 simple coumarins, 9 furanocoumarins, 17 acylquinic acids, all hydroxybenzoic- and hydroxicinnamic acid-—glycosides and sugar esters, and 18 flavonoids are reported for the first time in the species. The study is the first attempt at an in-depth characterization of angelols and numerous furanocoumarins by hyphenated technique UHPLC-Orbitrap-HRMS in both genera *Ostericum* and *Angelica*. It is worth noting that the fragmentation patterns of two subclasses angelols are suggested. The most prominent fragment ions of ESI-MS/MS spectra are depicted in Table 1, whereas thorough MS/MS data are included in Table S1. Extracted ion chromatograms with mass accuracy 5 ppm are presented in Figure 1, Figures S1 and S2. Taking into consideration that the MS/MS fragmentations of furanocoumarins have been thoroughly studied in several *Angelica* species, these data were used in our study for comparative purposes and cited accordingly. Identification confidence levels were according to the Çiçek et al., 2024 [15], and were as follows: Confirmed structure except for one or more stereochemical aspects (B); tentative identification matched with a standard compound, match of at least tR, MS and MS/MS with an actual authentic standard analyzed in parallel, preferably supported by other online data (C); tentative identification based on libraries, model compounds, etc. (D); relatively reliable evidence (D1); relatively poor evidence (D2) [15].

#### 2.1.1. Simple Coumarins

Herein, 28 simple coumarins substituted on the benzene ring with hydroxy, methoxy, prenyl, hydroxyalkyl, or epoxidized groups were annotated (Table 1 and Table S1, Figure 1A). Key points in the compound recognition (**2**, **4**, **6**, **11**, **21**, **22**, **24**) were fragment ions at *m*/*z* 147.044, 119.049, and 91.055, as has been observed in coumarin core [16].

**Figure 2:**
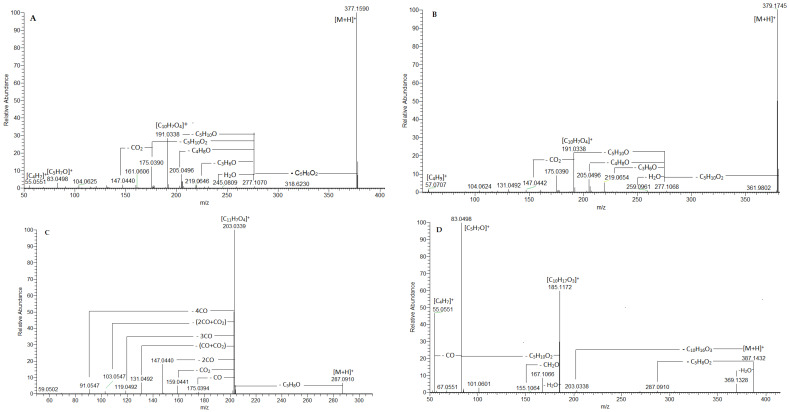
(-) ESI-MS/MS spectrum of angelol A, B, D, G, K (**14**) (**A**), angelol C, E, F, H (**15**) (**B**), oxypeucedanin (**46**) (**C**), ostruthol (**47**) (**D**).

Among the commonly found in the Apiaceae species hydroxycoumarins, ref. [14] umbelliferone (**3**) was identified by the prominent fragment ions at *m*/*z* 135.044 [M + H-CO]^+^, 107.050 [M + H-2CO]^+^ and 89.039 [M + H-2CO-H_2_O]^+^, while an additional methoxy group was deduced in scopoletin (**9**) from the transition 193.050 → 178.028 [M + H-CH_3_]^+^ (Table 1, Table S1). The presence of two methoxy groups was witnessed in hydroxy-dimethoxycoumarin isomers **7**, **8**, and **10** ([M + H]^+^ at *m*/*z* 223.060) by the subsequent losses of 15 Da at *m*/*z* 208.037 [M + H-CH_3_]^+^ and 193.013 [M + H-2CH_3_]^+^, corroborated by the ions at *m*/*z* 162.031 [M + H-CH_3_-H_2_O-CO]^+^ and 134.036 [M + H-CH_3_-H_2_O-2CO]^+^. An additional prenyl group was evidenced in isomers **22**/**24** at *m*/*z* 231.1015 [M + H]^+^ in comparison with **3**. This assignment was supported by the ions at *m*/*z* 175.039 [M + H-C_4_H_8_]^+^ and 119.049 [M + H-C_4_H_8_-2CO]^+^. Accordingly, the fragmentation pattern of **22**/**24** could be related to osthenol, previously reported in *O. palustre*/*A. pancicii* roots [6]. Likewise, osthenol-hexoside (**11**) was deduced by the prominent fragment ions at *m*/*z* 231.101 [M + H-C_6_H_10_O_5_]^+^ and 175.039. Regarding **26**, typical losses of prenyl moiety (C_5_H_8_) were registered at *m*/*z* 177.055 and 121.065 [M + H-C_5_H_8_-2CO]^+^, as has been seen in osthol, formerly isolated from *A. archangelica* roots [17]. The fragmentation pattern of **21** was delineated by the following transitions: 261.112 → 189.055 [M + H-C_4_H_8_O]^+^ → 161.060 [M + H-C_4_H_8_O-CO]^+^ → 133.065 [M + H-C_4_H_8_O-2CO]^+^ → 105.071 [M + H-C_4_H_8_O-3CO]^+^. Thus, epoxy-pentanyl moiety was deduced in **21**. It was assigned as meranzin, previously isolated from *A. sylvestris* leaves [18]. Six compounds (**12**–**14**, **16**–**18**) shared the same precursor ion [M + H]^+^ at *m*/*z* 377.159 (consistent with C_20_H_25_O_7_) (Table 1, Table S1). They were ascribed to the angelol-type coumarins characterized by 6-acyloxy-dihydroxy-3-methylbutyl, 7-methoxycoumarin backbone, formerly isolated from *A. pubescens* roots [19,20] and tentatively identified in *O. palustre*/*A. pancicii* roots [6]. The subsequent losses of 100.052 Da (C_5_H_8_O_2_) at *m*/*z* 277.107 and 102.068 Da (C_5_H_10_O_2_) at *m*/*z* 175.039 pointed out a methylbutenoic acid (angelic or tiglic acid) and dihydroxymethylbutyl functional group, respectively, as has been observed in Angelol A, B, D, G, K and N from *A. pubescens* [19,20]. This assignment was corroborated by the transitions *m*/*z* 175.039 → 147.044 (-CO), 175.039 → 131.049 (-CO_2_), 175.039 → 91.055 (-3CO) (Table S1, Figure 2A). Thus, the aforementioned isomers refer to the first angelol-type compounds. It is worth noting that angelol-isomers arise from the stereoisomerism at C-12 (R and S isomers) alongside the acyl C-5 groups—angeloyl (in angelol A, G, K) or tigloyl (in angelol B and D). In addition, a migration of the acyl group between C-11 and C-12 was reported, which gives a rise in isomeric pairs [19]. On the other hand, MS/MS spectra of the second angelol-type compounds **15**, **19**, and **20** with [M + H]^+^ at *m*/*z* 379.175 (consistent with C_20_H_27_O_7_) were acquired (Table 1 and Table S1, Figure 2B). They afforded an indicative ion at *m*/*z* 277.106 resulting from the loss of 102.068 Da consistent with either isovaleryl or 2-methylbutyl moiety, as has been seen in angelol C, E, F, H, I, and L from *A. pubescens* [19]. Afterward, the presence of angelol H and I has been confirmed in *A. dahurica* stem and root [21,22]. Concerning **25** with [M + H]^+^ at *m*/*z* 359.148, a methylbutenoic acid was deduced by the transition 359.148 → 259.0963 [M + H-C_5_H_8_O_2_]^+^, while the loss of 154.099 Da (C_9_H_14_O_2_) at *m*/*z* 205.049 indicated either epoxy-pentanyl or hydroxymethylbutenyl residue linked to the coumarin skeleton. Compound **28** at *m*/*z* 367.227 was assigned as umbelliprenin, formerly isolated from *O. palustre*/*A. pancicii* fruits [7].

**Table 1:** Secondary metabolites in *Ostericum palustre* methanol-aqueous extracts.

No.	Identified/Tentatively Annotated Compound	Molecular Formula	Exact Mass [M + H]^+^	Fragmentation Pattern in (+) ESI-MS/MS	t_R_ (min)	Δ ppm	Distribution	Level of Confidence	References
	**Simple coumarins**								
1.	dihydroxycoumarin-hexosoide ^b^	C_15_H_16_O_9_	341.0867	341.0863 (5.4), 179.0338 (100), 123.0442 (9.3)	2.70	−1.100	2	D1	
2.	hydroxy-(trihydroxypentanyl)-coumarin1 ^b^	C_14_H_16_O_6_	281.1020	281.1015 (100), 245.0806 (30.8), 175.0389 (14.5)	2.98	−1.655	1, 2	D1	
3.	umbelliferone	C_9_H_6_O_3_	163.0390	163.0389 (100), 135.0441 (57.0), 89.0391 (45.8)	3.19	−0.091	1, 2	D1	[6]
4.	hydroxy-(trihydroxypentanyl)-coumarin2 ^b^	C_14_H_16_O_6_	281.1020	281.1015 (100), 245.0806 (32.3), 175.0389 (16.2)	3.29	−1.546	1, 2	D1	
5.	dihydroxycoumarin^b^	C_9_H_6_O_4_	179.0339	179.0338 (100), 133.0285 (14.8), 123.0442 (25.2)	3.44	−0.588	2, 3	D1	
6.	hydroxy-(trihydroxypentanyl)-coumarin3 ^b^	C_14_H_16_O_6_	281.1020	281.1016 (100), 245.0806 (35.4), 175.0390 (16.3)	4.21	−1.440	1	D1	
7.	hydroxy-dimethoxycoumarin 1 ^b^	C_11_H_10_O_5_	223.0601	223.0660 (100), 190.0260 (9.2), 162.0311 (11.2)	4.51	−0.582	1	D1	
8.	hydroxy-dimethoxycoumarin 2 ^b^	C_11_H_10_O_5_	223.0601	223.0660 (100), 190.0260 (11.7), 162.0311 (10.6)	4.83	−0.582	1	D1	
9.	scopoletin	C_10_H_8_O_4_	193.0495	193.0495 (100), 178.0280 (14.3), 133.0284 (23.5)	5.04	−0.131	1, 2	D1	[6]
10.	hydroxy-dimethoxycoumarin 3 ^b^	C_11_H_10_O_5_	223.0601	223.0600 (100), 190.0261 (10.6), 162.0312 (11.8)	5.33	−0.314	1	D1	
11.	osthenol-hexoside ^b^	C_20_H_24_O_8_	393.1544	393.1539 (9.8), 231.1014 (100), 175.0390 936.9)	7.45	−1.257	1, 2	D1	
12.	angelol isomers 1 (A, B, D, G, K)	C_20_H_24_O_7_	377.1595	377.1589 (100), 205.0496 (7.5), 191.0338 (22.8)	9.19	−1.616	2, 3	D1	[6,19]
13.	angelol isomers 2 (A, B, D, G, K)	C_20_H_24_O_7_	377.1595	377.1588 (100), 205.0495 (7.7), 191.0338 (27.8)	9.39	−1.775	2, 3	D1	[6,19]
14.	angelol isomers (A, B, D, G, K) 3	C_20_H_24_O_7_	377.1595	377.1590 (100), 205.0496 (8.4), 191.0338 (29.8)	9.55	−1.351	1, 2, 3	D1	[6,19]
15.	angelol isomers 1 (C, E, F, H, I)	C_20_H_26_O_7_	379.1751	379.1745 (100), 205.0495 (6.4), 191.0338 (18.7)	9.73	−1.740	3	D1	[6,19]
16.	angelol isomers 4 (A, B, D, G, K)	C_20_H_24_O_7_	377.1595	377.1588 (100), 219.0651 (38.2), 205.0495 (12.2)	9.79	−1.510	2, 3	D1	[6,19]
17.	angelol isomers 5 (A, B, D, G, K)	C_20_H_24_O_7_	377.1595	377.1588 (100), 219.0651 (9.8), 205.0495 (12.2)	9.91	−1.934	3	D1	[6,19]
18.	angelol isomers 6 (A, B, D, G, K)	C_20_H_24_O_7_	377.1595	377.1589 (100), 219.0651 (35.9), 205.0495 (10.2)	10.05	−1.510	1, 2, 3	D1	[6,19]
19.	angelol isomers 2 (C, E, F, H, I)	C_20_H_26_O_7_	379.1751	379.1745 (100), 219.0651 (22.2), 205.0496 (10.2)	10.11	−1.740	1, 2, 3	D1	[6,19]
20.	angelol isomers 3 (C, E, F, H, I)	C_20_H_26_O_7_	379.1751	379.1746 (100), 205.0499 (8.5), 191.0338 (8.1)	10.26		3	D1	[6,19]
21.	methoxy-(hydroxymethylbutenyl)-coumarin (meranzin) ^b^	C_15_H_16_O_4_	261.1121	261.1119 (100), 189.0546 (15.8), 161.0597 (16.8)	10.35	−0.979	1, 2	D1	
22.	hydroxy-prenylcoumarin 1	C_14_H_14_O_3_	231.1016	231.1015 (11.4), 175.0390 (100), 147.0440 (15.8)	10.62	−0.307	1	D1	[6,19]
23.	coumarin ^b^	C_9_H_6_O_3_	147.0441	147.0440 (100), 119.0494 (15.4), 91.0548 (51.9)	11.32	−0.313	1	D1	
24.	hydroxy-prenylcoumarin 2	C_14_H_14_O_3_	231.1016	231.1015 (100), 175.0390 (62.2), 119.0494(7.1)	12.01	−0.350	1, 2	D1	[6]
25.	methylbutenyl-hydroxymethylbutenylcoumarin ^b^	C_20_H_22_O_6_	359.1489	359.1482 (100), 259.0963 (13.9), 205.0494 (28.4)	13.93	−2.102	1, 2, 3	D1	
26.	pentenyl-scopoletin ^b^	C_15_H_16_O_4_	261.1121	261.1104 (2.1), 193.0496 (100), 137.0597 (9.4)	14.60	−1.257	1	D1	
27.	methoxy-prenylcoumarin (osthol) ^b^	C_15_H_16_O_3_	245.1172	245.1171 (100), 177.0547 (3.1), 121.0650 (1.1)	14.88	−0.697	1, 2	D1	[17]
28.	umbelliprenin	C_24_H_30_O_3_	367.2268	367.2266 (100), 189.0548 (8.3), 163.0390 (78.7)	22.39	−0.211	1, 2, 3	D1	[7]
	**Furanocoumarins**								
29.	dihydroxy-dihydrofuranocoumarin 1 ^b^	C_14_H_14_O_5_	263.0914	263.0912 (73.5), 245.0806 (100), 217.0859 (28.4)	4.04	−0.798	1, 2, 3	D1	[16]
30.	dihydroxy-dihydrofuranocoumarin 2 ^b^	C_14_H_14_O_5_	263.0914	263.0913 (100), 245.0808 (20.6), 203.0703 (14.2)	4.63	−0.456	1, 2, 3	D1	[16]
31.	psoralen/angelicin	C_11_H_6_O_3_	187.0390	187.0389 (100), 143.0491 (8.8), 131.0491 (24.6)	4.93	−0.324	1, 2, 3	D1	[6]
32.	dihydroxy-dihydrofuranocoumarin 3 ^b^	C_14_H_14_O_5_	263.0914	263.0911 (100), 245.0806 (18.6), 203.0702 (14.6)	5.17	−1.178	1, 2, 3	D1	[16]
33.	marmesin/columbianetin ^b^	C_14_H_14_O_4_	247.0965	247.0963 (100), 229.0858 (15.0), 175.0390 (20.8)	5.61	−0.710	1, 2, 3	D1	[16]
34.	psoralen/angelicin	C_11_H_6_O_3_	187.0390	187.0390 (100), 131.0493 924.1), 115.0546 (17.4)	5.96	0.104	1, 2, 3	D1	[6]
35.	oxypeucedanin hydrate/heraclenol/aviprin	C_16_H_16_O_6_	305.1017	305.1016 (90.5), 203.0339 (100), 147.0441 (18.6)	6.42	−1.326	1, 2, 3	D1	[6,7,23]
36.	marmesin/columbianetin ^b^	C_14_H_14_O_4_	247.0963	247.0963 (100), 229.0858 (11.8), 175.0390 (18.5)	7.21	−0.589	1, 2, 3	D1	[16]
37.	oxypeucedanin hydrate/heraclenol/aviprin	C_16_H_16_O_6_	305.1017	305.1017 (82.2), 203.0338 (100), 147.0440 (22.6)	7.68	−1.031	1, 2, 3	D1	[6,7,23]
38.	xanthotoxol/bergaptol	C_11_H_6_O_4_	203.0339	203.0338 (100), 175.0389 (17.2), 91.0548 (4.5)	8.40	−0.567	1, 2, 3	D1	[6]
39.	byakangelicin/isobyakangelicin	C_17_H_18_O_7_	335.1125	335.1122 (100), 233.0443 (47.0), 218.0209 (19.7)	8.92	−1.132	1, 2, 3	D1	[6]
40.	byakangelicin/isobyakangelicin	C_17_H_18_O_7_	335.1125	335.1122 (100), 233.0444 (45.5), 218.0209 (19.7)	9.05	−1.043	1, 2, 3	D1	[6]
41.	bergapten/xanthotoxin ^b^	C_12_H_8_O_4_	217.0495	217.0494 (100), 202.0311 (40.7), 174.0311 (10)	9.83	−0.715	1, 3	D1	[17,24,25]
42.	Isopimpinellin ^a,b^	C_13_H_10_O_5_	247.0601	247.0600 (100), 232.0365 (23.9), 217.0131 (57.8)	9.89	−0.364	1, 2, 3	D1	[25]
43.	oxypeucedanin/pangelin/heraclenin/pabulenol/isogosferol/isooxypeucedanin	C_16_H_14_O_5_	287.0914	287.0910 (100), 229.0494 (32.3), 203.0339 (19.4)	10.14	−0.400	1, 3	D1	[6,7,25,26]
44.	oxypeucedanin/pangelin/heraclenin/pabulenol/isogosferol/isooxypeucedanin	C_16_H_14_O_5_	287.0914	287.0911 (56.2), 203.0338 (100), 147.0440 (25.9)	10.37	−0.310	1, 2, 3	D1	[7,23]
45.	Byakangelicol ^b^	C_17_H_16_O_6_	317.1020	317.1020 (13.5), 233.0443 (100), 218.0208 (20.0)	11.23	−0.015	1, 2, 3	D1	[16,23]
46.	oxypeucedanin/pangelin/heraclenin/pabulenol/isogosferol/isooxypeucedanin	C_16_H_14_O_5_	287.0914	287.0910 (82.3), 203.0338 (100), 147.0440 (18.9)	11.32	−1.498	1, 2, 3	D1	[7,23]
47.	ostruthol	C_21_H_22_O_7_	387.1438	387.1432 (18.4), 287.091 (1.0), 203.034 (1.2)	13.00	−1.729	1, 2, 3	D1	[6]
48.	xanthotoxol/bergaptol	C_11_H_6_O_4_	203.0339	203.0338 (100), 175.0390 (7.8), 147.0440 (37.03)	13.37	−0.370	1, 2, 3	D1	[6]
49.	Imperatorin ^a^	C_16_H_14_O_4_	271.0965	271.0967 (1.4), 203.0338 (100), 147.0440 (30.0)	13.37	−0.647	1, 2, 3	B	[6,7,23]
50.	phellopterin/knidilin	C_17_H_16_O_5_	301.1071	301.1066 (2.3), 233.0443 (100), 218.0209 (35.3)	13.96	−5.038	1, 2, 3	D1	[6,25,27]
51.	5-hydroxyxanthotoxin ^b^	C_12_H_8_O_5_	233.0444	233.0443 (100), 218.0208 (48.2), 162.0311 (7.8)	13.96	−0.719	1, 3	D1	[14]
52.	phellopterin/knidilin ^b^	C_17_H_16_O_5_	301.1071	301.1061 (1.8), 233.0443 (100), 218.0209 (14.4)	14.38	−5.337	1, 2, 3	D1	[12,13]
53.	xanthotoxol/bergaptol	C_11_H_6_O_4_	203.0339	203.0338 (100), 147.0440 (23.7), 131.0492 (8.0)	14.53	−0.587	1, 2, 3	D1	[6]
54.	isoimperatorin ^a^	C_16_H_14_O_4_	271.0965	271.0964 (0.6), 203.0339 (100), 147.0440 (30)	14.53	−2.307	1, 2, 3	B	[6,7,23]
55.	angeloylpangelin	C_21_H_20_O_6_	369.1333	369.1328 (3.9), 269.0805 (6.9), 167.1066 (76.8), 83.0498 (100), 55.0551 (48.2)	16.24	−0.515	1, 2, 3	D1	[7]
**No.**	**Identified/Tentatively Annotated Compound**	**Molecular Formula**	**Exact Mass** **[M − H]^−^**	**Fragmentation Pattern in (-) ESI-MS/MS**	**t_R_** **(min)**	**Δ ppm**	**Distribution**	**Level of Confidence**	**References**
	**Acylquinic acids**								
56.	neochlorogenic acid ^a,b^	C_16_H_18_O_9_	353.0867	353.0877 (46.4), 191.0551 (100), 179.0340 (64.2)	2.36	−0.185	1, 2, 3	B	
57.	chlorogenic acid ^a^	C_16_H_18_O_9_	353.0867	353.0878 (5.6), 191.0551 (100), 85.0279 (5.2)	3.19	0.070	1, 2, 3	B	[6,10]
58.	4-caffeoylquinic acid ^b^	C_16_H_18_O_9_	353.0867	353.0879 (36.2), 179.0339 (65.9), 173.0443 (100)	3.38	0.155	1, 2, 3	D1	
59.	3-feruloylquinic acid ^b^	C_17_H_20_O_8_	367.1034	367.1033 (0.9), 193.0498 (100), 134.0360 (54.0)	3.45	0.505	1, 2, 3	D1	
60.	5-caffeoylquinic acid isomer ^b^	C_16_H_18_O_9_	353.0867	353.0881 (6.6), 191.0550 (100), 85.0279 (8.3)	3.89	0.835	1, 2, 3	D1	
61.	5-*p*-coumaroylquinic acid 1 ^b^	C_16_H_18_O_8_	337.0928	337.0929 (10.2), 191.0552 (100), 85.0279 (4.6)	3.97	0.384	1, 2, 3	D1	
62.	5-feruloylquinic acid 1 ^b^	C_17_H_20_O_8_	367.1034	367.1022 (17.5), 191.0550 (100), 93.0330 (30.1)	4.41	−0.587	1, 2, 3	D1	
63.	5-*p*-coumaroylquinic acid 2 ^b^	C_16_H_18_O_8_	337.0928	337.0929 (7.7), 191.0549 (100), 85.0278 (7.4)	4.61	0.117	1, 2, 3	D1	
64.	5-feruloylquinic acid 2 ^b^	C_17_H_20_O_8_	367.1034	367.1033 (9.5), 191.0552 (100), 85.0279 (7.5)	4.91	−0.587	2, 3	D1	
65.	3, 4-dicaffeoylquinic acid ^a,b^	C_25_H_24_O_12_	515.1189	515.1197 (100), 173.0446 (53.6), 135.0439 (41.4)	5.68	0.370	1, 2, 3	B	
66.	3, 5-dicaffeoylquinic acid ^b^	C_25_H_24_O_12_	515.1189	515.1194 (20.0), 353.0878 (97.7), 191.0550 (100)	5.86	−0.232	1, 2, 3	D1	
67.	1, 5-dicaffeoylquinic acid ^a,b^	C_25_H_24_O_12_	515.1189	515.1192 (33.4), 353.0878 (88.0), 191.0551 (100)	6.02	0.581	2, 3	B	
68.	4, 5-dicaffeoylquinic acid ^b^	C_25_H_24_O_12_	515.1189	515.1191 (100), 179.0340 (64.7), 173.0445 (94.4)	6.23	−0.697	2, 3	D1	
69.	3-caffeyl−5-*p*-coumaroylquinic acid ^b^	C_25_H_24_O_11_	499.1251	499.1243 (28.9), 353.0879 (59.4), 191.0553 (100)	6.58	−0.630	1, 2, 3	D1	
70.	3-feruloyl-5-caffeoylquinic acid ^b^	C_26_H_26_O_12_	529.1356	529.1321(10.0), 367.1032 (84.1), 193.0497 (100)	6.82	−5.687	1, 2, 3	D1	
71.	3-caffeoyl-5-feruloylquinic acid ^b^	C_26_H_26_O_12_	529.1356	529.1348(52.4), 353.0878(44.9), 191.0551 (100)	6.89	−0.717	1, 2	D1	
72.	4-caffeyl-5-*p*-coumaroylquinic acid ^b^	C_25_H_24_O_11_	499.1251	499.1249 (100), 353.0878 (46.9), 173.0445 (90.9)	6.98	0.712	1, 2, 3	D1	
73.	4-caffeyl-5-feruloylquinic acid ^b^	C_26_H_26_O_12_	529.1356	529.1371 (100), 353.0878 (54.6), 173.0445 (98.3)	7.18	3.781	1, 2, 3	D1	
	**Hydroxybenzoic and hydroxycinnamic acids**								
74.	protocatechuic acid-*O*-hexoside ^b^	C_13_H_16_O_9_	315.0727	315.0722 (100), 152.0103 (58.0), 108.0202 (87.2)	1.72	0.269	1, 2, 3	D1	
75.	protocatechuic acid ^a,b^	C_7_H_6_O_4_	153.0182	153.0181 (19.3), 109.0280 (100), 81.0330 (2.0)	2.03	−7.855	1, 2, 3	B	
76.	hydroxybenzoyl-*O*-hexose ^b^	C_13_H_16_O_8_	299.0778	299.0774 (100), 179.0341 (46.4), 137.0230 (89.6), 93.0330 (19.0)	2.06	0.399	1	D1	
77.	*p*-hydroxyphenylacetic acid *O*-hexoside ^b^	C_14_H_18_O_8_	313.0929	313.0931 (0.7), 151.0388 100), 107.0487 (98.6)	2.15	−1.427	1, 2, 3	D1	
78.	protocatechuic acid *O*-pentosylhexoside ^b^	C_18_H_24_O_13_	447.1144	447.1142 (100), 152.0103 (45.5), 108.0202 (32.7)	2.37	−0.545	1, 2	D1	
79.	caffeic acid-*O*-hexoside 1 ^b^	C_15_H_18_O_9_	341.0871	341.0852 (5.3), 179.0340 (19.6), 135.0438 (56.1)	2.43	−7.609	1, 2, 3	D1	
80.	caffeic acid-*O*-hexoside 2 ^b^	C_15_H_18_O_9_	341.0871	341.0848 (1.9), 179.0341 (1.9), 135.0438 (100)	2.53	8.349	2, 3	D1	
81.	vanillyl-*O*-hexose ^b^	C_14_H_18_O_9_	329.0875	329.0880 (100), 209.0446 (30.1), 167.0339 (53.8)	2.48	0.531	1, 2, 3	D1	
82.	4-hydroxybenzoic acid ^a,b^	C_7_H_6_O_3_	137.0230	137.0231 (100), 108.0202 (7.7), 93.0331 (4.8)	2.84	−9.614	1, 2, 3	B	
83.	3-hydroxybenzoic acid ^a,b^	C_7_H_6_O_3_	137.0230	137.0231 (21.6), 93.0330 (100), 65.0380 (0.6)	2.95	−9.9.6	1, 2, 3	B	
84.	hydroxybenzoic acid-*O*-hexoside ^b^	C_13_H_16_O_8_	299.0778	299.0768 (1.8), 137.0230 (100), 93.0330 (41.3)	2.99	−1.641	1, 2, 3	D1	
85.	caffeic acid-*O*-hexoside 3 ^b^	C_15_H_18_O_9_	341.0871	341.0870 (13.8), 179.0340 (14.2), 135.0438 (100)	2.93	−2.244	2, 3	D1	
86.	caffeic acid-*O*-hexoside 4 ^b^	C_15_H_18_O_9_	341.0871	341.0875 (30.8), 179.0340 (100), 135.0438 (64.1)	3.07	−1.012	1, 2, 3	D1	
87.	quinic acid ^b^	C_7_H_12_O_6_	191.0549	191.0553 (100), 173.0446 (1.4), 85.0279 (18.3)	3.19	−4.404	1, 2, 3	D1	
88.	caffeic acid-*O*-hexoside 5 ^b^	C_15_H_18_O_9_	341.0871	341.0874 (13.1), 179.0340 (100), 135.0438 (75.1)	3.27	−1.276	1, 2	D1	
89.	coumaric acid-*O*-hexoside ^b^	C_15_H_18_O_8_	325.0930	325.0918 (1.5), 163.0390 (100), 119.0488 (80.5)	3.34	−3.263	1, 2, 3	D1	
90.	caffeic acid ^a,b^	C_9_H_8_O_4_	179.0339	179.0340 (20.5), 135.0438 (100), 107.0488 (1.3)	3.54	−5.764	1, 2, 3	B	
91.	vanillic acid-*O*-hexoside ^b^	C_14_H_18_O_9_	329.0875	329.0881 (2.1), 167.0339 (100), 123.0437 (21.0)	3.38	0.895	1, 2, 3	D1	
92.	*m*-coumaric acid ^a,b^	C_9_H_8_O_3_	163.0389	163.0390 (10.0), 119.0489 (100), 93.0330 (1.3)	4.56	−6.731	1, 2, 3	B	
93.	salicylic acid ^a,b^	C_7_H_6_O_3_	137.0230	137.0232 (14.3), 93.0330 (100), 65.0379 (1.5)	6.25	−9.176	1, 2, 3	B	
**Flavonoids**									
94.	naringenin di-*C*-hexoside ^b^	C_27_H_32_O_15_	595.1668	595.1667 (100), 385.0929 (30.8), 355.0824 (31.8)	3.65	−0.157	2	D1	
95.	luteolin -7-*O*-glucoside ^a,b^	C_21_H_19_O_11_	447.0934	447.0931 (100), 285.0403 (93.3), 133.0281 (13.1)	4.80	−0.458	2	B	
96.	eriodictyol *O*-rutinoside ^b^	C_27_H_32_O_15_	595.1668	595.1667 (97.4), 287.0561 (88.5), 151.0024 (100)	5.12	−0.157	1, 2, 3	D1	
97.	isoquercitrin ^a,b^	C_21_H_20_O_12_	463.0885	463.0883 (100), 300.0275 (79.3), 271.0248 (37.8)	5.19	0.131	1, 2, 3	B	
98.	luteolin –7-*O*-rutinoside^a^	C_27_H_30_O_15_	593.1512	593.1513 (100), 285.0404 (96.4), 133.0283 (4.7)	5.23	0.197	1, 2, 3	B	[26]
99.	hyperoside ^a,b^	C_21_H_20_O_12_	463.0885	463.0883 (100), 300.0276 (64.7), 271.0249 (29.3)	5.30	0.196	1, 2, 3	B	
100.	isorhamnetin 3-*O*-rutinoside ^a,b^	C_28_H_32_O_17_	623.1618	623.1611 (100), 315.0512 (74.6), 151.0025 (12.6)	5.53	−1.104	2, 3	B	
101.	naringenin 7-*O*-rutinoside ^b^	C_27_H_32_O_14_	579.1719	579.1718 (66.2), 271.0613 (100), 119.0488 (27.1)	5.76	−0.222	2, 3	D1	
102.	apigenin 7-*O*-rutinoside ^b^	C_27_H_30_O_14_	577.1563	577.1559 (47.5), 269.0456 (100), 117.0333 (3.7)	5.82	−0.587	1, 2, 3	D1	
103.	kaempferol 3-O-glucoside ^a,b^	C_21_H_19_O_11_	447.0934	447.0932 (100), 284.0327 (52.2), 227.0346 (33.3)	5.87	−0.189	2, 3	B	
104.	chrysoeriol/diosmetin *O*-hexoside ^b^	C_22_H_22_O_11_	461.1089	461.1089 (100), 299.0560 (78.0), 284.0327 (40.9)	5.93	−0.097	1, 2	D1	
105.	chrysoeriol/diosmetin *O*-rutinoside ^b^	C_28_H_32_O_15_	607.1668	607.1671 (75.4), 299.0560 (100), 284.0326 (63.2)	6.15	0.340	1, 2, 3	D1	
106.	isorhamnetin 3-*O*-glucoside ^a,b^	C_22_H_22_O_12_	477.1044	477.1036 (100), 314.0435 (29.0), 299.0197 (41.0)	6.16	−0.522	1, 2, 3	B	
107.	hesperetin *O*-rutinoside ^b^	C_28_H_34_O_15_	609.1825	609.1826 (55.1), 301.0714 (100), 151.0025 (6.7)	6.21	0.208	1, 2, 3	D1	
108.	quercetin ^a,b^	C_15_H_10_O_7_	301.0354	301.0353 (100), 221.0085 (24.9), 151.0024 (42.6)	7.62	−0.318	1, 2, 3	B	
109.	naringenin ^a,b^	C_15_H_12_O_5_	271.0612	271.0613 (100), 151.0025 (61.5), 119.0488 (48.7)	8.57	0.418	1, 2, 3	B	
110.	chrysoeriol ^a,b^	C_16_H_12_O_6_	299.0561	299.0560 (92.9), 284.6326 (100), 107.0124 (4.8)	8.97	−0.473	1, 2, 3	B	
111.	quercetin *O*-coumaroyldeoxyhexoside ^b^	C_30_H_26_O_13_	593.1301	593.1301 (100), 301.0348 (45.2), 255.0299 (12.6)	9.15	0.010	1, 2, 3	D1	
112.	quercetin *O*-dicoumaroyldeoxyhexoside ^b^	C_39_H_32_O_15_	739.1668	739.1670 (100), 301.0353 (79.7), 151.0024 (16.4)	11.46	0.198	1, 2, 3	D1	

^a^—Compare to reference standards; ^b^—Reported for the first time in species; Confidence level: B: Confirmed structure except for one or more stereochemical aspects; D: Tentative identification based on libraries, model compounds, etc.; D1: Relatively reliable evidence; 1—Roots extract; 2—Leaves extract; 3—Inflorescences extract.

#### 2.1.2. Furanocoumarins

Owing to the structural complexity (linear and angular furanocoumarins), regio- and stereo-isomers occur within the furanocoumarins [13,14].

Most of the furanocoumarins in *O. palustre* extracts occurred in several isomeric forms along the chromatograms (Figure 2B) and the trivial names of the annotated isobars ascribed to each protonated molecule [M + H]^+^ are depicted in Table 1 and Table S1. Typical ions of furanocoumarin fragmentation pathway were produced by neutral losses of CO (−28 Da), 2 CO (56 Da), 3 CO (−84 Da), CO_2_ (−44 Da), (CO + CO_2_) (72 Da) [16]. Exemplified by **31** and **34**, precursor ion [M + H]^+^ at *m*/*z* 187.0390 afforded a series of aforementioned neutral losses at *m*/*z* 159.044, 131.049, 103.055, 143.049, and 115.055, respectively, as has been observed in psoralen and its angular isomer angelicin [16] (Table S1). In this regards, psoralen has been previously tentatively identified in *O. palustre*/*A. pancicii* aerial parts [6]. The abovementioned fragments could be assigned as key points in furanocoumarins recognition. Additional a hydroxyl group in the furanocoumarin skeleton was denoted in **38**, **48**, and **53** ([M + H]^+^ at *m*/*z* 203.034) where subsequent losses of CO and CO_2_ resulted at fragment ions at *m*/*z* 175.039 [M + H-CO]^+^, 159.044 [M + H-CO_2_]^+^, 147.044 [M + H-2CO]^+^, 131.049 [M + H-CO-CO_2_]^+^, 119.049 [M + H-3CO]^+^, 103.055 [M + H-3CO-CO_2_]^+^, 91.055 [M + H-4CO]^+^, as has been seen in the isomeric pair xanthotoxol/bergaptol [16] (Table 1, Table S1). A methoxy group was evidenced in **41** ([M + H]^+^ at *m*/*z* 217.049) by the prominent ion at *m*/*z* 202.031 [M + H-CH_3_]^+^, while two methoxy groups were suggested in **42** ([M + H]^+^ at *m*/*z* 247.060) by the transitions 247.060 → 232.037 → 217.013 (Table 1). Thus, the former compound could be assigned as bergapten/xanthotoxin, while the latter was identified as isopimpinellin by comparison with a reference standard.

The fragmentation pathways of **49** and **54** were delineated by the following transitions: *m*/*z* 271.089 → 203.034 [M + H-C_5_H_8_]^+^ → 175.039 [M + H-C_5_H_8_-CO]^+^ → 147.044 [M + H-C_5_H_8_-2CO]^+^ → 103.055 [M + H-C_5_H_8_-2CO-CO_2_]^+^, as has been seen in imperatorin/isoimperatorin (substituent being lost first) [16,24]. Both furanocoumarins have been previously isolated from *O. palustre/A. pancicii* fruits and roots [6,7]. Based on the comparison with the retention times and fragmentation patterns of reference standards, compounds **49** and **54** were unambiguously identified as imperatorin and isoimperatorin, respectively.

Compounds **35** and **37** shared the same fragmentation patterns with **49** and **54** except for the occurrence of dihydroxyisopentanyl moiety (102.068 Da, C_5_H_10_O_2_) (instead isopentenyl residue C_5_H_8_, 68.062 Da). This assignment was supported by the prominent fragment ions at *m*/*z* 203.034 [M + H-C_5_H_10_O_2_]^+^ followed by the commonly found in furanocoumarins series of neutral losses (see **38** and **48**). In this respect, isobaric oxypeucedanin hydrate, heraclenol and aviprin have been isolated from *O. palustre/A. pancicii* and *O. grosseserratum* roots along with *A. sylvestris* fruits, and *A. dahurica* roots [7,9,13,24]. In the same manner **43**, **44,** and **46** afforded fragment ion at *m*/*z* 203.034 [M + H-C_5_H_8_O]^+^ suggesting the loss of C_5_H_8_O (84.057 Da) consistent with either hydroxyisopentenyl (pangelin, pabulenol (gosferol)) or epoxy-isopentanyl moiety (oxypeucedanin, heraclenin) [13] (Table 1 and Table S1, Figure 2C). This assignment is consistent with the occurrence of oxypeucedanin in the *O. palustre/A. pancicii* fruits and roots, and *O. grosseserratum* roots [6,7,28]. An additional methoxy group was deduced in **39, 40** ([M + H]^+^ at *m*/*z* 335.112), and **45** ([M + H]^+^ at *m*/*z* 317.102) in comparison with **35** and **37**, respectively, as have been observed in byakangelicin/isobyakangelicin and byakangelicol from *A. dahurica* roots and *O. grosseserratum* roots [21,23]. Likewise, the fragmentation patterns of **50** and **52** ([M + H]^+^ at *m*/*z* 301.107) were defined by the fragment ions at *m*/*z* 233.044 [M + H-C_5_H_8_]^+^ and 218.021 [M + H-C_5_H_8_-CH_3_]^+^. Thus, they could be related to the isomeric pair phellopterin/knidilin, formerly isolated from *A. archangelica* seeds [25] and *A. dahurica* roots [13]. The (+) ESI-MS/MS spectrum of **47** was consistent with ostruthol, formerly isolated from *O. palustre*/*A. pancicii* roots [6].

The prominent fragment ions at *m*/*z* 287.092 [M + H-C_5_H_8_O_2_]^+^ and 185.117 [C_10_H_17_O_3_]^+^ corroborated the annotated furanocoumarin (Table 1 and Table S1, Figure 2D). Concerning **55**, the presence of angeloyl (An) moiety was witnessed by the prominent fragment ions at *m*/*z* 269.081 [M + H-C_5_H_8_O_2_]^+^, 167.107 [C_10_H_15_O_2_]^+^, 83.050 [AnOH + H-H_2_O]^+^, and 55.055 [AnOH + H-H_2_O-CO]^+^. Accordingly, **55** could be assigned as angeloylpangelin, formerly isolated from *O. palustre*/*A. pancicii* fruits [6].

#### 2.1.3. Acylquinic Acids

Nine mono- and 9 diacylquinic acids (AQAs) were identified or annotated in the *O. palustre* extracts (Table 1 and Table S1, Figure S1). The key points of fragmentation patterns were delineated elsewhere [14,29]. Taking into consideration that the base peak was observed at *m*/*z* 191.055, **57**/**60**, **61**/**63,** and **62**/**64** were ascribed to 5-caffeoyl-, 5-*p*-coumaroyl, and 5-feruloylquinic acid isomeric pairs, respectively. The substitution at C-3 of the quinic acid framework was evidenced by the abundant ions at *m*/*z* 179.034 (**56**) and 193.050 (**59**), respectively (Table 1). diAQA refers to the following subclasses: dicaffeoylquinic acids (diCQA) at *m*/*z* 515.120 [M − H]^−^, (**65**–**68**), feruloul-caffeoylquinic acids (FCQA) at *m*/*z* 529.135 (**70**, **71** and **73**) and *p*-coumaroyl-caffeoylquinic acids (p-CoCQA) at *m*/*z* 499.125 (**69** and **72**) (Table S1). Vicinal diCQA 3, 4-diCQA (**65**), 4,5-diCQA (**68**), 4C-5-*p*-CoQA (**72**), and 4C-5FQA (**73**) were defined by the distinctive “dehydrated” peak at *m*/*z* 173.045 [quinic acid-H-H_2_O]^−^. Compounds **69** and **71** afforded prominent ion at *m*/*z* 353.088, indicating a loss of the *p*-coumaroyl (146 Da) and feruloyl (176 Da) moiety before the caffeoyl one. Furthermore, both compounds yielded a base peak at *m*/*z* 191.055, as has been seen in C-5-substituted ACAs alongside *m*/*z* 119.049 [*p*-coumaric acid-H-CO_2_]^−^ (**69**) and 134.036 [ferulic acid-H-CH_3_-CO_2_]^−^ (**71**) (Table 1). Thus, **69** and **71** were ascribed as 3C-5-*p*-CoCQA and 3C-5FQA, respectively. Neochlorogenic (**56**), chlorogenic (**57**), 3, 4-dicaffeoylquinic (**65**), and 1, 5-dicaffeoylquinic (**67**) acid were unambiguously identified by comparison with reference standards.

#### 2.1.4. Hydroxybenzoic and Hydroxycinnamic Acids and Derivatives

A variety of hydroxybenzoic and hydroxycinnamic acid glycosides including **74**, **77**–**80**, **84**–**86**, **88**, **89,** and **91** were annotated (Table 1 and Table S1). Ester bonds in hydroxybenzoyl hexoses **76** and **81** were evidenced by the hexose cross ring cleavages ^0,2^Hex (−120 Da), ^0,3^Hex (−90 Da), and ^0,4^Hex (−60 Da). Accordingly, **76** and **81** were assigned as hydroxybenzoyl-hexose and vanillyl-hexose, respectively. Based on the comparison with the retention times and fragmentation patterns of reference standards, protocatechuic (**75**), 4-hydroxybenzoic (**82**), 3-hydroxybenzoic (**83**), caffeic (**90**), *m*-coumaric (**92**), and salicylic (**93**) acid were unambiguously identified in the *O. palustre* extracts.

#### 2.1.5. Flavonoids

Overall, 7 flavonol glycosides (**97**, **99**, **100**, **103**, **106**, **111** and **112**), 5 flavone glycosides (**95**, **98**, **102**, **104** and **105**) and 4 flavanone glycosides (**94**, **96**, **101** and **107**) were dereplicated/annotated in the assayed *O. palustre* extracts (Table 1 and Table S1, Figure S2). Compounds **96**, **98**, **100**–**102**, **105,** and **107** were closely related to the same fragmentation pattern affording indicative fragment ions at 271.061 (naringenin), *m*/*z* 301.072 (hesperetin), 285.040 (luteolin), 315.051 (isorhamnetin), 299.056 (chrysoeriol) and 287.056 (eriodyctiol) [(M-H)-C_12_H_20_O_9_]^−^, respectively, suggesting rutinosides. Compounds **111** and **112** shared the same fragmentation pattern, delineated by the transitions 593.130 → 447.093 → 301.035 (**111**) and 739.167 → 593.131 → 301.035 (**112**) indicating the loss of coumaroyldeoxyhexose (C_15_H_16_O_6_, 292.095) and dicoumaroyldeoxyhexose (C_24_H_22_O_8_, 438.132 Da), respectively. Accordingly, the aforementioned compounds were annotated as quercetin- coumaroyldeoxyhexoside (**111**) and dicoumaroyldeoxyhexoside (**112**). Compounds luteolin -7-*O*-glucoside (**95**), isoquercitrin (**97**), luteolin -7-*O*-rutinoside (**98**), hyperoside (**99**), isorhamnetin 3-*O*-rutinoside (**100**), kaempferol 3-*O*-glucoside (**103**), isorhamnetin 3-*O*-glucoside (**106**), quercetin (**108**), naringenin (**109**), and chrysoeriol (**110**) were identified on the base of comparison with reference standards.

#### 2.1.6. Total Phenolic and Flavonoid Content

Spectrophotometric methods were used to investigate the total phenolic and flavonoid content in the tested extracts. The results are summarized in Table 2. The inflorescences had the highest level of phenolic content, with a value of 33.8 mg GAE/g dry extract, followed by leaves (22.1 mg GAE/g) and roots (19.0 mg GAE/g). The total flavonoid levels in leaves and inflorescences were very similar, while the lowest level was found in the roots (1.28 mg RE/g). Apparently, the levels of total phenolics and flavonoids changed depending on which part of the plant was used. Similar to our findings, the roots of most plants contained lower levels of total bioactive compounds than the leaves and flowers [20,21,22]. Due to their pollination and protection mechanisms, the aerial parts of most plants produce higher levels of secondary metabolites. Several researchers have reported different levels of total phenolic content in members of the *Angelica* genus in the literature [23,24]. Our results reveal a lower total phenolic and flavonoid content in comparison with those previously reported by Mileski et al. (2017), where the aerial parts contain 143.99 mg GAE/g dry ethanol extract and 35.15 mg QE/g methanol extract [6].

However, spectrophotometric results do not fully reflect the actual levels. In particular, the Folin–Ciocalteu reagent can be reduced by some phytochemicals, including peptides; therefore, the results obtained may be inaccurate [23].

#### 2.1.7. Antioxidant Effects

In the current study, we examined the antioxidant properties of *O. palustre* extracts using several assays, including radical scavenging (ABTS and DPPH), reducing power (CUPRAC and FRAP), phosphomolybdenum, and metal chelation. The results are shown in Table 2. The ABTS and DPPH assays assess the ability of antioxidant compounds to quench free radicals and are based on hydrogen donation. In both assays, inflorescences exhibited the strongest radical scavenging ability (DPPH: 47.9 mg TE/g; ABTS: 61.8 mg TE/g). However, the roots exhibited the weakest DPPH radical scavenging ability and the leaves exhibited the lowest ABTS radical scavenging ability. The electron-donation ability of antioxidants is considered an important mechanism for stopping chain-breaking reactions. To this end, we performed CUPRAC and FRAP assays to measure the electron-donation ability of the extracts that were tested. As with radical scavenging ability, the highest reducing ability was found in the inflorescences extract in both assays (CUPRAC: 102.2 mg TE/g; FRAP: 57.4 mg TE/g). When the results of the radical scavenging and reducing assays were evaluated together, they showed a similar trend to that observed for the total phenolic content in the extracts. Thus, we can infer that phenolic components are the main contributors to the ability to donate hydrogen and electrons. Several researchers have also reported a strong correlation between total phenolic content and radical scavenging/reducing abilities, in accordance with our findings [30,31,32]. The phosphomolybdenum assay involves reducing Mo (VI) to Mo (V) using antioxidants, resulting in a blue molybdate-phosphate complex that can be detected at 695 nm. As can be seen in Table 2 the roots extract demonstrated the greatest ability with 1.72 mmol TE/g in the phosphomolybdenum assay, followed by inflorescences and leaves. Different results in the phosphomolybdenum assay can be explained by the presence of non-phenolic compounds (tocopherols, vitamin C, etc.) and their actions in the extracts. Chelation of transition metals, including iron and copper, is an important mechanism relating to the management of the most dangerous free radical, the hydroxyl radical, in the Fenton reaction. The inflorescences extract exhibited the best metal chelation ability with 20.1 mg EDTAE/g, and the other extracts exhibited almost the same ability. Overall, the inflorescences extract demonstrated the greatest activity in almost all antioxidant assays, except for the phosphomolybdenum assay. The radical scavenging activity of *O. palustre*/*A. pancicii* has been previously studied. The aerial parts ethanol extract and leaves hexane extract showed the highest activity in the DPPH assay with IC_50_ 0.26 mg/mL and 1.2 mg/mL, respectively [6,7]. In comparison, the essential oil had a lower activity in this assay with IC_50_ > 10 mg/mL. The roots aqueous extract actively scavenged ABTS radicals (IC_50_ 0.64 mg vit. C equivalents/g extract), whereas aqueous extract from aerial parts possessed the most pronounced activity with IC_50_ 2.45 mg/mL against lipid peroxydation in the β-carotene bleaching assay in comparison with methanol and ethanol extracts [6]. Previous investigations reported the biological activity of *O. palustre*/*A. pancicii* essential oil [5,7]. In a study of Stankovic et al. [33] the 80% methanolic extract of *O. palustre*/*A. pancicii* was examined for its antioxidant properties using the DPPH, ABTS, and FRAP methods, and it was found to exhibit moderate antioxidant ability. Lately, the antioxidant activity of *O. grosseserratum* root ethylacetate extract in TEAC, ORAC, and DPPH assays has been reported [34]. As can be seen in Table 2, the observed antioxidant ability can be explained by the presence of certain compounds in the study. The presence of coumarins, flavonoids and phenolic acids in particular can be attributed to the observed antioxidant ability in the current study [35,36,37].

#### 2.1.8. Enzyme Inhibitory Effects

Herein, we examined the enzyme inhibitory effects of *O. palustre* extracts against cholinesterase, amylase, glucosidase and tyrosinase (Table 2). The best AChE inhibition was found in the roots extract with 2.12 mg GALAE/g, but this was not statistically different from leaves extract (2.09 mg GALAE/g). Regarding BChE inhibition, leaves and inflorescences extracts had similar inhibitory effects, but interestingly the roots were not active in BChE. In the anti-tyrosinase ability, leaves and inflorescences extracts displayed almost similar abilities, but the roots had a weaker ability by comparison. It should be noted that mushroom tyrosinase, although widely used in preliminary screening assays, may not fully reflect the inhibitory behavior against human tyrosinase due to structural and catalytic differences [38]. Therefore, the present results should be interpreted with caution, and further studies using human-relevant models are warranted.

Regarding the amylase and glucosidase inhibitory effects, the tested extracts exhibited almost the same activities (amylase: 0.33–0.38 mmol ACAE/g; glucosidase: 1.57–1.64 mmol ACAE/g). Collagenase, elastase, and hyaluronidase inhibition potentials of extracts from different parts of the plant were compared via the IC_50_ values obtained (Table 3). The inhibitory effects against collagenase, elastase, and hyaluronidase were evaluated using full dose–response curves, and IC_50_ values were calculated by non-linear regression analysis. Epigallocatechin gallate (EGCG) was used as a positive control for collagenase and elastase inhibition, while tannic acid was used as a reference inhibitor for hyaluronidase. These reference compounds are widely employed in enzyme inhibition studies involving natural products. Statistical comparisons between extracts and reference inhibitors were performed using one-way ANOVA, and all differences were found to be significant (*p* ≤ 0.0001). The roots extract exhibited a good inhibitory effect by showing the lowest IC_50_ values for all three enzymes (collagenase: 37.22 µg/mL; elastase: 42.47 µg/mL; hyaluronidase: 32.09 µg/mL). The leaves extract showed moderate activity; IC_50_ values were determined as 61.27 µg/mL, 72.11 µg/mL, and 54.75 µg/mL, respectively. The weakest inhibition was observed in the inflorescences extract, which had the highest IC_50_ values for all enzymes (collagenase: 93.62 µg/mL; elastase: 100 µg/mL; hyaluronidase: 80.60 µg/mL). There is limited information in the literature on the enzyme-inhibiting properties of *O. palustre* extracts. We found a few papers on this topic, but they were about the essential oil of this plant. Lately, prominent anti-acetylcholinesterase activity has been reported for the *O. palustre/A. pancicii* fruit hexane extract and essential oil with IC_50_ 1.62 and 1.90 mg/mL, respectively. In the context of the effects on the nervous system, furanocoumarins such as imperatorin, phellopterin, and simple coumarin scopoletin have been found to exert protective effects by ameliorating memory disturbances and mitigating anxiety-like behaviors [13]. *O. grosseserratum* root extract (70% ethanol) has been reported to exert anti-diabetic and anti-obesity effects by increasing the expression of genes related to glucose uptake in adipocytes [10]. At 0.4 mg/mL plant extract exerted 41% α-glucosidase inhibitory activity ascribed to chlorogenic acid occurrence. Indeed, *Angelica dahurica* roots have been used in the traditional medicine for the treatment of skin diseases [13]. New studies support the claim about this application. Imperatorin and isoimperatorin inhibit melanogenesis through the inhibition of the tyrosinase biosynthesis in B16 melanoma cells which emphases their potential as a whitening agent in cosmetics [39]. Recently, the anti-wrinkle properties of *Angelica gigas* root extracts in in vitro and clinical trials were evaluated. The extracts induced the expression of type I collagen in a dose-dependent manner without cytotoxicity, and demonstrated statistically significant anti-wrinkle effects after 8 weeks as compared with the placebo [40].

The observed enzyme inhibitory effects of *O. palustre* extracts can be attributed to their chemical profiles. For example, furanocoumarins and phenolic compounds may contribute to the extracts’ observed enzyme inhibitory abilities. In particular, coumarin derivatives, chlorogenic acid and flavonoids have been identified as natural inhibitors on the tested enzyme in previous studies [41,42,43]. In this point, *O. palustre* can be considered as a source of natural enzyme inhibitors in health-promoting applications.

#### 2.1.9. Supervised Sparse Partial Least Squares Discriminant Analysis

**Figure 3:**
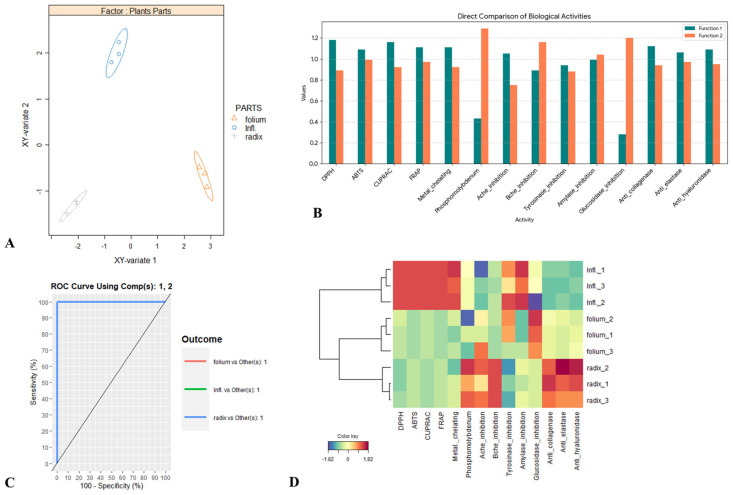
sPLS-DA analysis on biological activities of *Ostericum palustre* considering the parts as class membership (Roots, Leaves, Inflorescences). (**A**) Samples plots. (**B**) Most discriminant biological activities evaluation by VIP approach. (**C**) AUC average and ROC curve using one-vs-all comparisons. (**D**) Clustered Image Maps analysis.

The dataset was submitted to supervised sparse partial least squares discriminant analysis (sPLS-DA) to determine diagnostic fluctuation in biological activities considering the studied three parts of *Angelica*, and the results are presented in Figure 3.

Figure 3A illustrates the two-dimensional score plot of the model, revealing a clear separation among the three plant parts. In particular, roots and leaves were distinctly discriminated from inflorescences along the first function. The variables contributing most strongly to this discrimination were DPPH, ABTS, CUPRAC, FRAP, metal-chelating activity, AChE inhibition, anti-collagenase, anti-elastase, and anti-hyaluronidase activities (Figure 3B). Likewise, roots and inflorescences were clearly differentiated from leaves along the second function. The observed segregation on this function was primarily explained by four biological activities including phosphomolybdenum activity, BChE inhibition, amylase inhibition, and glucosidase inhibition (Figure 3B). The performance of the discriminant model was subsequently evaluated. Analysis of the receiver operating characteristic (ROC) curves revealed an area under the curve (AUC) of 1.0 for both functions, indicating a complete separation among the three plant parts (Figure 3C). Finally, examination of the heatmap revealed distinct activity patterns among the plant parts. Inflorescences extracts displayed the strongest antioxidant and amylase inhibitory activities. Roots exhibited the highest anti–skin-enzyme activities, whereas leaves were characterized by the greatest phosphomolybdenum activity as well as the highest levels of both cholinesterase inhibitory activities.

**Figure 4:**
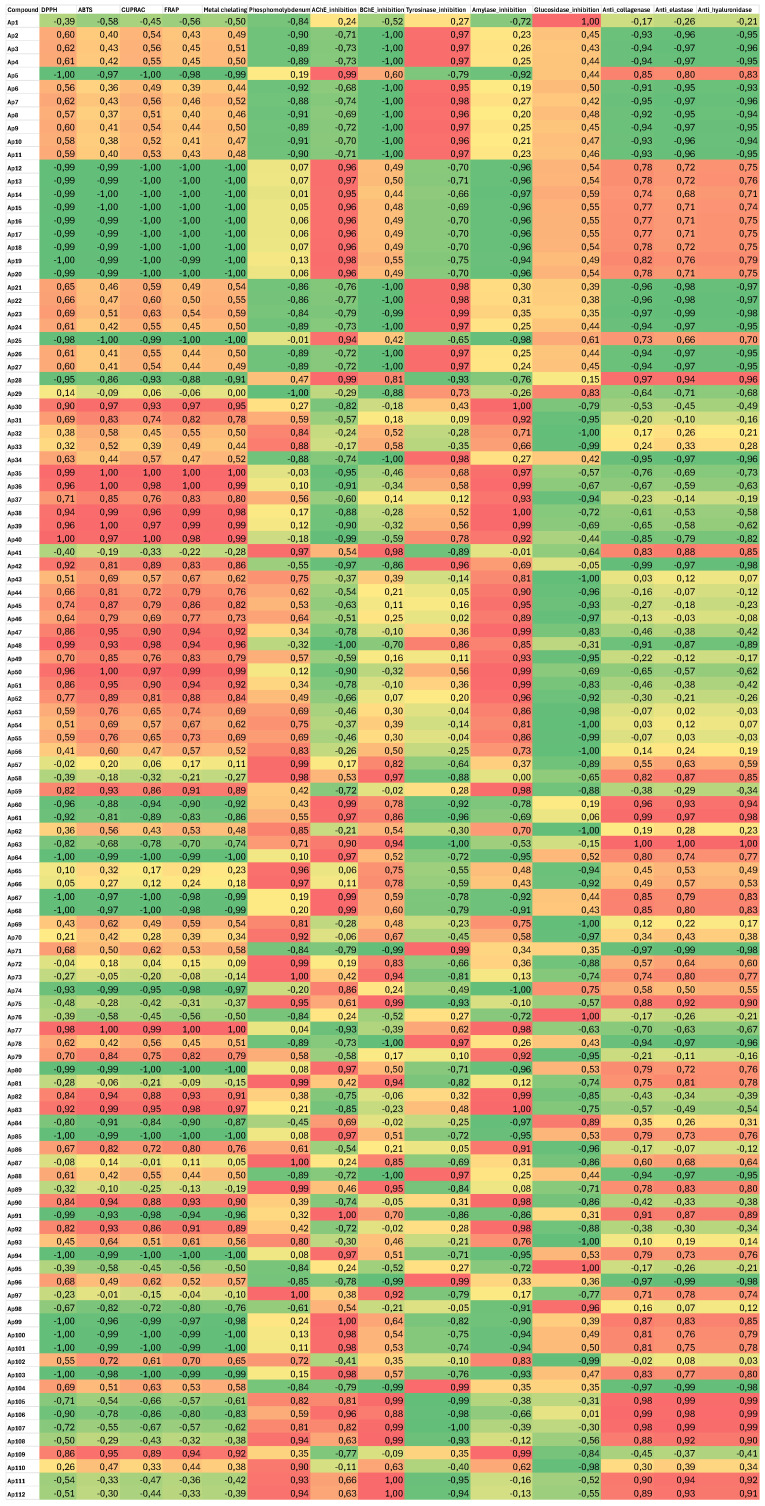
Hypothesized role of the molecules in the observed biological effects according to the Pearson correlation test. A threshold of ±0.80 was established to identify significant correlations.

Finally, the hypothesized role of the molecules in the observed biological effects is evaluated and the results presented in Figure 4 and Table 4. Pearson relationship analysis reveals numerous correlation coefficients exceeding the threshold of ±0.80. Among the 112 molecules studied, 42 (37%) exhibit a profile of priority interest. This group notably includes 15 potent antioxidants capable of neutralizing free radicals and 21 compounds displaying a remarkable neuroprotective profile. Furthermore, 12 molecules stand out for their ability to preserve the skin’s extracellular matrix, while 6 act specifically on digestive enzymes. It is worth noting that 6 of these candidates are multifunctional, combining several major biological activities.

## 3. Materials and Methods

### 3.1. Plant Material

*Ostericum palustre* (*Angelica pancicii*) roots and aerial parts were collected near to Kumata hut, Vitosha Mts (42.594346′ N 23.250671′ E, altitude 1724 m a.s.l.), during the full flowering stage, in August 2024 (data about the habitat are provided in the Supplementary Material). The plant material was identified by Assoc. Prof. Reneta Gevrenova according to [2]. A voucher specimen was deposited at the Herbarium Academiae Scientiarum Bulgariae (SOM 179647). Plant samples were separated into roots, leaves, and inflorescences and dried at room temperature (20–22 °C) and 50% of relative humidity. The plant materials were dried until they reached a constant weight.

### 3.2. Sample Extraction

Air-dried powdered roots (14.4 g), leaves (20.0g), and inflorescences (22.6 g) were extracted with 50% MeOH (1:30 *w*/*v*) by ultrasound (100 kHz, ultra-sound bath Biobase UC-20C, Jinan, China) for 15 min, three times, at room temperature. The methanol was evaporated in vacuo and water residue was lyophilized (lyophilizer Biobase BK-FD10P) to yield the crude extract of roots (2.5 g), leaves (0.94 g), and inflorescences (4.74 g). Then, the lyophilized extracts were dissolved in 50% methanol (0.1 mg/mL), filtered through a 0.45 μm syringe filter and were subjected to UHPLC–HRMS analyses. The same extract was used for pharmacological tests.

### 3.3. Chemicals

Acetonitrile (for LC–MS), formic acid (for LC–MS), and methanol (for HPLC) were purchased from Honeywell (Charlotte, NC, USA). The reference standards were obtained from Extrasynthese (Genay, France) (for protocatechuic, 4-hydroxybenzoic, 3-hydroxybenzoic, caffeic, *m*-coumaric, and salicylic acid, luteolin -7-*O*-glucoside, isoquercitrin, luteolin -7-*O*-rutinoside, hyperoside, isorhamnetin 3-*O*-rutinoside, kaempferol 3-*O*-glucoside, isorhamnetin 3-*O*-glucoside, quercetin, chrysoeriol), Phytolab (Vesten-bergsgreuth, Bavaria, Germany) (chlorogenic acid, 1,5-dicaffeoylquinic acid, 3,4-dicaffeoylquinic acid, naringenin, isopimpinellin, and isoimperatorin, and Sigma-Aldrich (Saint Louis, MO 63103, USA) (pimpinellin).

The chemicals were purchased from Sigma-Aldrich (Darmstadt, Germany). They were: 2,2′-azino-bis(3-ethylbenzothiazoline-6-sulphonic acid (ABTS), 1,1-diphenyl-2-picrylhydrazyl (DPPH), gallic acid, rutin, electric eel acetylcholinesterase (AChE) (type-VI-S, EC 3.1.1.7), horse serum butyrylcholinesterase (BChE) (EC 3.1.1.8), galantamine, acetylthiocholine iodide (ATChI), butyrylthiocholine chloride (BTChI), 5,5-dithio-bis(2-nitrobenzoic) acid (DTNB), tyrosinase (EC1.14.18.1, mushroom), glucosidase (EC. 3.2.1.20, from *Saccharomyces cerevisiae*), amylase (EC. 3.2.1.1, from porcine pancreas), sodium carbonate, Folin–Ciocalteu reagent, hydrochloric acid, sodium hydroxide, trolox, ethylenediaminetetraacetate (EDTA), neocuproine, cupric chloride, ammonium acetate, ferric chloride, 2,4,6-Tris(2-pyridyl)-s-triazine (TPTZ), ammonium molybdate, ferrozine, ferrous sulphate hexahydrate, kojic acid, and acarbose. All chemicals were of analytical grade. Additionally, the following chemicals, commercially available from Sigma-Aldrich (Saint Louis, MO, USA), were used in enzyme assays related to skin enzymes: Collagenase, Type I, Clostridium histolyticum (E.C. 3.4.24.3) (1.1 U/mL), Porcine pancreatic elastase (E.C. 3.4.21.36), (3.33 mg/mL), and Hyaluronidase Type I-S, from bovine testes (E.C. 3.2.1.35).

### 3.4. UHPLC-HRMS

The UHPLC-HRMS analyses were performed as previously described [29] on Q Exactive Plus mass spectrometer (ThermoFisher Scientific, Inc., Waltham, MA, USA) with a heated electrospray ionization (HESI-II) probe, in negative and positive ion modes within the *m*/*z* range from 150 to 1500. The chromatographic separation was achieved on a reversed phase column C18 (1.8 µm, 2.1 × 100 mm), and the temperature was 40 °C. The mobile phase consisted of 0.1% formic acid in water (A) and 0.1% formic acid in acetonitrile (B), the run time was 33 min; the flow rate and other chromatographic conditions were previously described. Xcalibur 4.2 (ThermoScientific, Waltham, MA, USA) and MZ mine 2.0 software were used for data processing.

### 3.5. Assay for Total Phenolic and Flavonoid Contents

Total phenolics and flavonoids quantification was carried out according to the methods previously described [44]. Gallic acid (GA) and rutin (RE) served as standards in the assays, and the outcomes were reported as gallic acid equivalents (GAE) and rutin equivalents per gram extract. The experimental details are given in the Supplementary Materials.

### 3.6. Assays for In Vitro Antioxidant Capacity

The antioxidant activity of the studied extract was evaluated [44]. The DPPH, ABTS radical scavenging, CUPRAC, and FRAP test results were presented as milligrams of Trolox equivalents (TE) per gram of extract. The antioxidant potential determined by the phosphomolybdenum (PBD) assay was measured in millimoles of Trolox equivalents (TE) per gram of extract, and metal chelating activity (MCA) was conveyed as milligrams of disodium edetate equivalents (EDTAE) per gram of extract. The experimental details are given in the Supplemental Materials.

### 3.7. Inhibitory Effects Against Some Key Enzymes

Enzyme inhibition experiments on the samples were conducted following established protocols [44]. Amylase and glucosidase inhibition were quantified in mmol acarbose equivalents (ACAE) per gram of extract, while acetylcholinesterase (AChE) and butyrylcholinesterase (BChE) inhibition were expressed in milligrams of galanthamine equivalents (GALAE) per gram of extract. Tyrosinase inhibition was measured in milligrams of kojic acid equivalents (KAE) per gram of extract. The experimental details are given in the Supplementary Materials. The collagenase, elastase, and hyaluronidase inhibitory effects were evaluated as IC_50_ values, as reported in our earlier study [45].

### 3.8. Statistical Analysis

All experiments were conducted in triplicate, and differences among extracts were evaluated for statistical significance. One-way ANOVA was performed in GraphPad Prism (version 9.2), followed by Tukey’s post hoc test for pairwise comparisons. Statistical significance was set at *p* < 0.05. Subsequently, the data was scaled and submitted to sPLS-DA (sparse partial least squares discriminant analysis) and CIMs (clustered image maps) analysis to discriminate the samples in terms of plant’s parts. To identify the highest discriminant biological activities, only variable with variable importance in projection (VIP) scores greater than 1 were retained. Afterward, a Pearson correlation test was performed to assess molecular contributions to the biological outcomes. R statistical software (v. 4.2.3) was used for the analysis.

## 4. Conclusions

The present study allowed for a thorough comparative phytochemical profiling of Bulgarian *O. palustre* methanol-aqueous extracts from roots, leaves, and inflorescences. Herein, more than 110 secondary metabolites, including simple coumarins and furanocoumarins, acylquinic acids analogs, flavonoids, hydroxybenzoic and hydroxycinnamic acid derivatives were annotated/dereplicated by means of UHPLC-Orbitrap-HRMS. Among them, 14 simple coumarins including a series of angelols, 9 furanocoumarins, 17 acylquinic acids, all hydroxybenzoic- and hydroxicinnamic acid—glycosides and sugar esters, and 18 flavonols, flavones, and flavanones are reported for the first time. Taking into consideration the lack of comprehensive profiling of another *Ostericum* species, mainly native to Asia, we are not able to draw chemophenetic conclusions. To understand the relationship between plant parts and biological activity, discriminant analysis (sPLS-DA) was performed. The strongest antioxidant potential (DDPH, ABTS, FRAP, CUPRAC and metal chalating) of the inflorescences extract was related to the furanocoumarins substituted with hydroxy, methoxy, (angeloyl-)-dihydroxyisopentanyloxy and isopentenyloxy functional groups. The leaves extract would support an antioxidant response and inhibit cholinesterases. Thus, our results highlighted their neuroprotective activity associated with a series of angelols, feruloyl- and dicaffeoylquinic acids alongside caffeic acid-hexosides and well-known flavonoids hyperoside, isorhamnetin 3-*O*-rutinoside, naringenin 7-*O*-rutinoside, and kempferol 3-*O*-glucoside. It is noteworthy that the roots extract exerted anti-collagenase, anti-elastase and anti-hyaluronidase activity, involved in the regulation of the skin barrier. Hydroxy-, methoxy- isopentenyl- and epoxyisopentenyl derivatives of coumarin accounted for the root extract anti-aging activity. The study argues for in vitro cytotoxicity tests (such as MTT or resazurin-based assays) and in vivo experimental models with *O. palustre* extracts designed to evaluate the safety profile, antioxidant status, and conditions associated with skin aging.

## Figures and Tables

**Table 2:** Total bioactive components, antioxidant properties, and enzyme inhibitory effects of the tested extracts.

*Total Bioactive Compounds*	*Roots*	*Leaves*	*Inflorescences*
Total phenolic content (mg GAE/g)	19.0 ± 0.3 ^c^	22.1 ± 0.2 ^b^	33.8 ± 0.1 ^a^
Total flavonoid content (mg RE/g)	1.28 ± 0.05 ^c^	7.98 ± 0.04 ^a^	7.77 ± 0.15 ^b^
***Antioxidant activity***			
DPPH scavenging ability (mg TE/g)	28.1 ± 0.1 ^c^	30.8 ± 0.2 ^b^	47.9 ± 0.1 ^a^
ABTS scavenging ability (mg TE/g)	37.3 ± 0.3 ^b^	34.3 ± 0.3 ^c^	61.8 ± 0.5 ^a^
CUPRAC (mg TE/g)	60.0 ± 0.9 ^c^	62.5 ± 0.2 ^b^	102.2 ± 0.7 ^a^
FRAP (mg TE/g)	36.7 ± 0.4 ^b^	35.1 ± 0.2 ^c^	57.4 ± 0.4 ^a^
Metal chelating (mg EDTAE/g)	16.4 ± 0.7 ^b^	16.4 ± 0.4 ^b^	20.1 ± 0.3 ^a^
Phosphomolybdenum (mmol TE/g)	1.72 ± 0.04 ^a^	1.42 ± 0.08 ^c^	1.55 ± 0.01 ^b^
***Enzyme inhibitory properties***			
AChE inhibition (mg GALAE/g)	2.12 ± 0.03 ^a^	2.09 ± 0.04 ^a^	2.01 ± 0.02 ^b^
BChE inhibition (mg GALAE/g)	n.a	2.49 ± 0.02 ^a^	2.50 ± 0.03 ^a^
Tyrosinase inhibition (mg KAE/g)	56.8 ± 0.3 ^b^	59.0 ± 1.2 ^a^	59.7 ± 0.7 ^a^
Amylase inhibition (mmol ACAE/g)	0.35 ± 0.01 ^ab^	0.33 ± 0.01 ^b^	0.38 ± 0.01 ^a^
Glucosidase inhibition (mmol ACAE/g)	1.58 ± 0.01 ^b^	1.64 ± 0.01 ^a^	1.57 ± 0.04 ^b^

Values are reported as mean ± SD of three parallel measurements. GAE: Gallic acid equivalent; RE: Rutin equivalent; TE: Trolox equivalent; EDTAE: EDTA equivalent; GALAE: Galanthamine equivalent; KAE: Kojic acid equivalent; ACAE: Acarbose equivalent; Different letters indicate significant differences in plant parts (*p* < 0.05).

**Table 3:** The determination of the inhibitory effects of the tested extracts on related enzymes to elucidate their role in the regulation of the skin barrier and anti-aging effects.

Extracts	Anti-Collagenase IC_50_ (µg/mL)	Anti-Elastase IC_50_ (µg/mL)	Anti-Hyaluronidase IC_50_ (µg/mL)
Roots	37.22 ± 1.65 *	42.47 ± 4.01 *	32.09 ± 3.17 *
Leaves	61.27 ± 3.28 *	72.11 ± 1.94 *	54.75 ± 2.95 *
Inflorescences	93.62 ± 4.96 *	100 ± 7.87 *	80.60 ± 5.03 *
EGCG	21.26 ± 2.06 *	28.15 ± 2.81 *	-
Tannic acid	-	-	25.08 ± 1.11 *

*p*-values obtained from the statistical analyses (n = 3) of IC_50_ values of extracts obtained from different parts of the plant compared with reference inhibitors such as EGCG and Tannic Acid were presented. * was indicated *p* ≤ 0.0001.

**Table 4:** Overview of significant correlation coefficients (r ≥ |0.80|).

Biological Target	Key Compounds	Biological Activity Test Concerned	Total Compounds Exceeding the ±0.80 Correlation Threshold
**Antioxidants**	Ap30, Ap35, Ap36, Ap38, Ap39, Ap40, Ap47, Ap48, Ap50, Ap51, Ap77, Ap82, Ap83, Ap90, Ap92	DPPH/ABTS/FRAP/CUPRAC	15 compounds
**Neuroprotectors**	Ap5, Ap12-20, Ap25, Ap64, Ap67, Ap68, Ap80, Ap85, Ap94, Ap99-101, Ap103	AChE/BChE + anti-antioxidants	21 compounds
**Anti-age**	Ap2, Ap3, Ap4, Ap6-11, Ap 21-24	Anti-collagenase/Anti-elastase/Anti-hyaluronidase	12 compounds
**Digestive enzymes**	Ap30, Ap36, Ap38, Ap39	Amylase/Glucosidase	6 compounds

## Data Availability

The original contributions presented in the study are included in the article; further inquiries can be directed to the corresponding authors.

## Supplementary Materials

The following supporting information can be downloaded at: https://www.mdpi.com/article/10.3390/plants15081172/s1, Figure S1: EIC of acylquinic acids proceed with mass tolerance of 5 ppm as follows: 56–58 and 60 at *m*/*z* 353.0867 (353.0849–353.0885); 59, 62, and 64 at *m*/*z* 367.1034 (367.1016–367.1052); 61 and 63 at *m*/*z* 337.0928 (337.0911–337.0945); 65–68 at *m*/*z* 515.1189 (515.1163–515.1215); 69 and 72 at *m*/*z* 499.1251 (499.1226–499.1276); 70, 71, and 73 at *m*/*z* 529.1356 (529.1330–529.1382); Figure S2: EIC of flavonoids proceed with mass tolerance of 5 ppm as follows: 94 at *m*/*z* 595.1668 (595.1638–595.1698); 95 at *m*/*z* 447.0934 (447.0912–447.0956); 96 at *m*/*z* 595.1668 (595.1638–595.1698); 97 at *m*/*z* 463.0885 (463.0862–463.0908); 98 at *m*/*z* 593.1512 (593.1482–593.1542); 99 at *m*/*z* 463.0885 (463.0862–463.0908); 100 at *m*/*z* 623.1618 (623.1587–623.1649); 101 at *m*/*z* 579.1719 (579.1690–579.1748); 102 at *m*/*z* 577.1563 (577.1534–577.1592); 103 at *m*/*z* 447.0934 (447.0912–447.0956); 104 at *m*/*z* 461.1089 (461.1066–461.1112); 105 at *m*/*z* 607.1668 (607.1638–607.1698); 106 at *m*/*z* 477.1044 (477.1020–477.1068); 107 at *m*/*z* 609.1825 (609.1795–609.1855); 108 at *m*/*z* 301.0354 (301.0339–301.0369); 109 at *m*/*z* 271.0612 (271.0598–271.0626); 110 at *m*/*z* 299.0561 (299.0548–299.0576); 111 at *m*/*z* 593.1301 (593.1271–593.1331); 112 at *m*/*z* 739.1668 (739.1631–739.1705); Table S1: MS/MS data of annotated compounds. References [46,47,48] are cited in the Supplementary Materials.
