# Supplementary material for: Advancing the Potential of Ostericum palustre (Besser) Besser (Synonym Angelica pancicii Vandas ex. Velen.) of Bulgarian Origin as a Source of Bioactive Compounds: Metabolite Profiling and Pharmacological Activity
Source: Plants (Basel). 2026 Apr 10;15(8):1172. doi: 10.3390/plants15081172 (PMC13120622; doi:10.3390/plants15081172)
Supplement: Supplementary file 1 [file plants-15-01172-s001.zip › plants-4219918-supplementary/Suppl_Mat rev.pdf]

Advancing the potential of *Ostericum palustre* (Besser) Besser (synonym *Angelica pancicii* Vandas ex. Velen.) of Bulgarian origin as a source of bioactive compounds: metabolite profiling and pharmacological activity

Reneta Gevrenova *[1], Gokhan Zengin [2], Kouadio Ibrahime Sinan [2,3] , Inci Kurt-Celep[4], Alexandra Stefanova[5], and Dimitrina Zheleva-Dimitrova [1,*]

[1] Department of Pharmacognosy, Faculty of Pharmacy, Medical University-Sofia, 1000 Sofia, Bulgaria; rgevrenova@pharmfac.mu-sofia.bg (R.G.); dzheleva@pharmfac.mu-sofia.bg (D.Z);
[2] Physiology and Biochemistry Research Laboratory, Department of Biology, Science Faculty, Selcuk University, Konya 42130, Turkey; gokhanzengin@selcuk.edu.tr (G.Z.), sinankouadio@gmail.com (KIS)
[3] Faculty of Biological Sciences, Department of Biochemistry-Genetics, Educational and Research Unit of Genetic, University of Peleforo Gon Coulibaly (UPGC),Korhogo, BP 1328, Côte d'Ivoire.
[4] Department of Pharmaceutical Biotechnology, Faculty of Pharmacy, İstanbul Okan University, Tuzla, İstanbul 34940, Türkiye, inci.celep@okan.edu.tr (IKC)
[5] Department of Pharmacology, pharmacotherapy, and toxicology, Faculty of Pharmacy, Medical University, 1000 Sofia, Bulgaria; astefanova22@gmail.com (A.S.);

* Correspondence: dzheleva@pharmfac.mu-sofia.bg (D.Z); rgevrenova@pharmfac.mu-sofia.bg (R.G.);

Regarding Fig. 1 (main document)

Extracted ion chromatogram (EIC) of simple coumarins proceed with mass tolerance of 5 ppm as follows:
1 at *m/z* 341.0867 (341.0850-341.0884); **2** at *m/z* 281.1020 (281.1006-281.1034); **3** at *m/z* 163.0340 (163.0332-163.0348); **4** at *m/z* 281.1020 (281.1006-281.1034); **5** at *m/z* 179.0339 (179.0330-179.0348); **6** at *m/z* 281.1020 (281.1006-281.1034); **7** at *m/z* 223.0601 (223.0590-223.0612); **8** at *m/z* 223.0601 (223.0590-223.0612); **9** at *m/z* 193.0495 (193.0485-193.0505); **10** at *m/z* 223.0601 (223.0590-223.0612); **11** at *m/z* 393.1544 (393.1524-393.1564); **12-14** at *m/z* 377.1595 (377.1576-377.1614); **15** at *m/z* 379.1751 (379.1732-379.1770); **16-18** at *m/z* 377.1595 (377.1576-377.1614); **19 -20** at *m/z* 379.1751 (379.1732-379.1770); **21** at *m/z* 261.1121 (261.1108-261.1134); **22** at *m/z* 231.1016 (231.1004-231.1028); **23** at *m/z* 147.0441 (147.0434-147.0448; **24** at *m/z* 231.1016 (231.1004-231.1028); **25** at *m/z* 359.1489 (359.1471-359.1507); **26** at *m/z* 261.1121 (261.1108-261.1134); **27** at *m/z* 245.1172 (245.1160-245.1184); **28** at *m/z*367.2268 (367.2250-367.2286).

EIC of furanocoumarins proceed with mass tolerance of 5 ppm as follows:
29 at *m/z* 263.0914 (263.0901-263.0927); **30** at *m/z* 263.0914 (263.0901-263.0927); **31** at *m/z* 187.0390 (187.0381-187.0399); **32** at *m/z* 263.0914 (263.0901-263.0927); **33** at *m/z* 247.0963 (247.0953-247.0977); **34** at *m/z* 187.0390 (187.0381-187.0399); **35** at *m/z* 305.1017 (305.1002-305.1032); **36** at *m/z* 247.0963 (247.0953-247.0977); **37** at *m/z* 305.1017 (305.1002-305.1032); **38** at *m/z* 203.0339 (203.0329-203.0349); **39** at *m/z* 335.1125 (335.1108-335.1142); **40** at *m/z* 335.1125 (335.1108-335.1142); **41** at *m/z* 217.0495 (217.0484-217.0506); **42** at *m/z* 247.0601 (247.0589-247.0613); **43** at *m/z* 287.0914 (287.0900-287.0928); **44** at *m/z* 287.0914 (287.0900-287.0928); **45** at *m/z* 317.1020 (317.1004-317.1036); **46** at *m/z* 287.0914 (287.0900-287.0928); **47** at *m/z* 387.1438 (387.1419-387.1457); **48** at *m/z* 203.0339 (203.0329-203.0349); **49** at *m/z* 271.0965 (271.0951-271.0979); **50** at *m/z* 301.1071 (301.1056-301.1086); **51** at *m/z* 233.0444 (233.0432-233.0456); **52** at *m/z* 301.1071 (301.1056-301.1086); **53** at *m/z* 203.0339 (203.0329-203.0349); **54** at *m/z* 271.0965 (271.0951-271.0979); 55 at *m/z* 369.1333 (369.1315-369.1351).

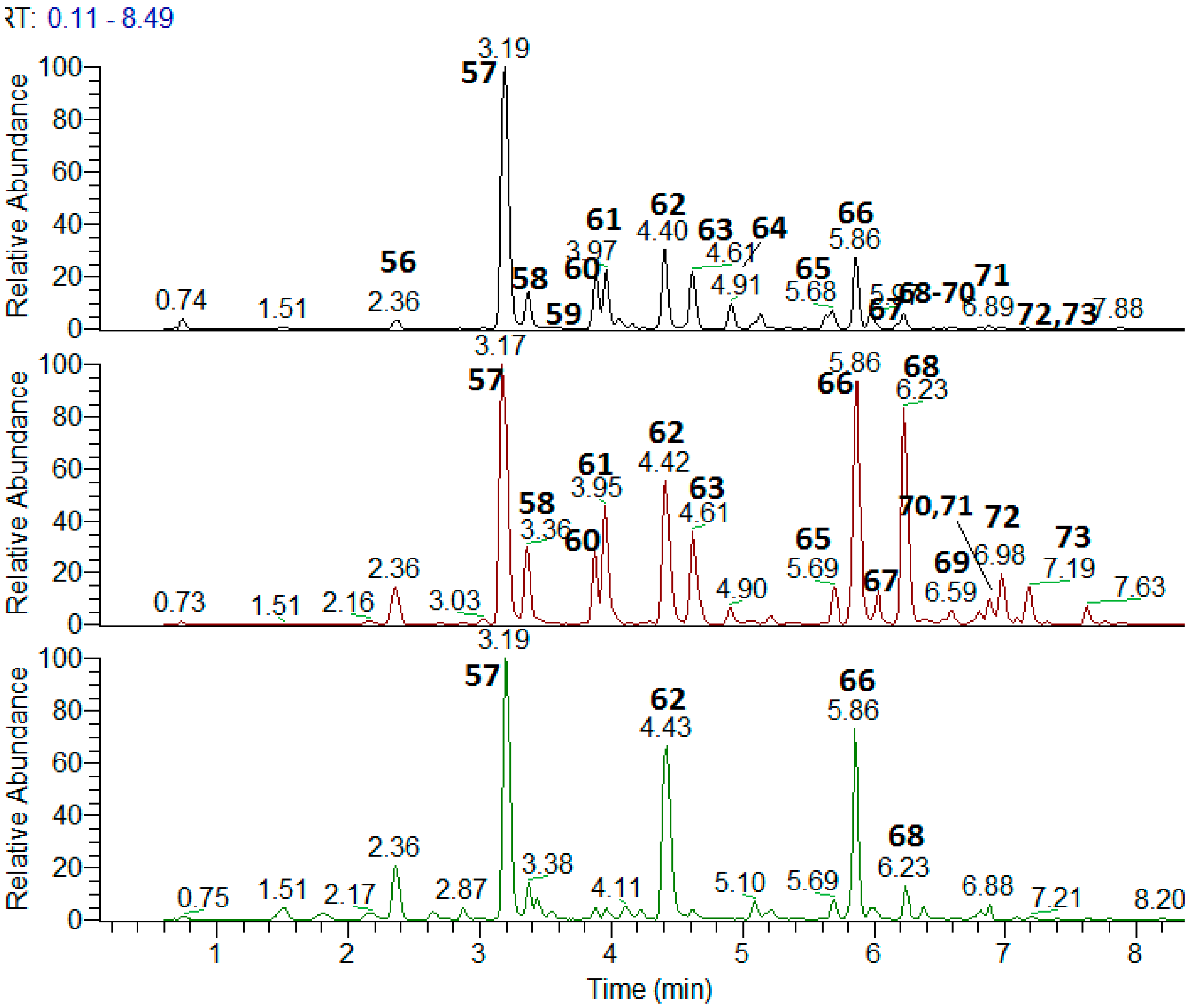

Figure S1. EIC of acylquinic acids proceed with mass tolerance of 5 ppm as follows: **56-58** and **60**at *m/z* 353.0867 (353.0849-353.0885); **59, 62** and **64** at *m/z* 367.1034 (367.1016-367.1052); **61** and **63** at *m/z* 337.0928 (337.0911-337.0945); **65-68** at *m/z* 515.1189 (515.1163-515.1215); 69 and 72 at *m/z* 499.1251 (499.1226-499.1276); 70, 71 and 73 at *m/z* 529.1356 (529.1330-529.1382).

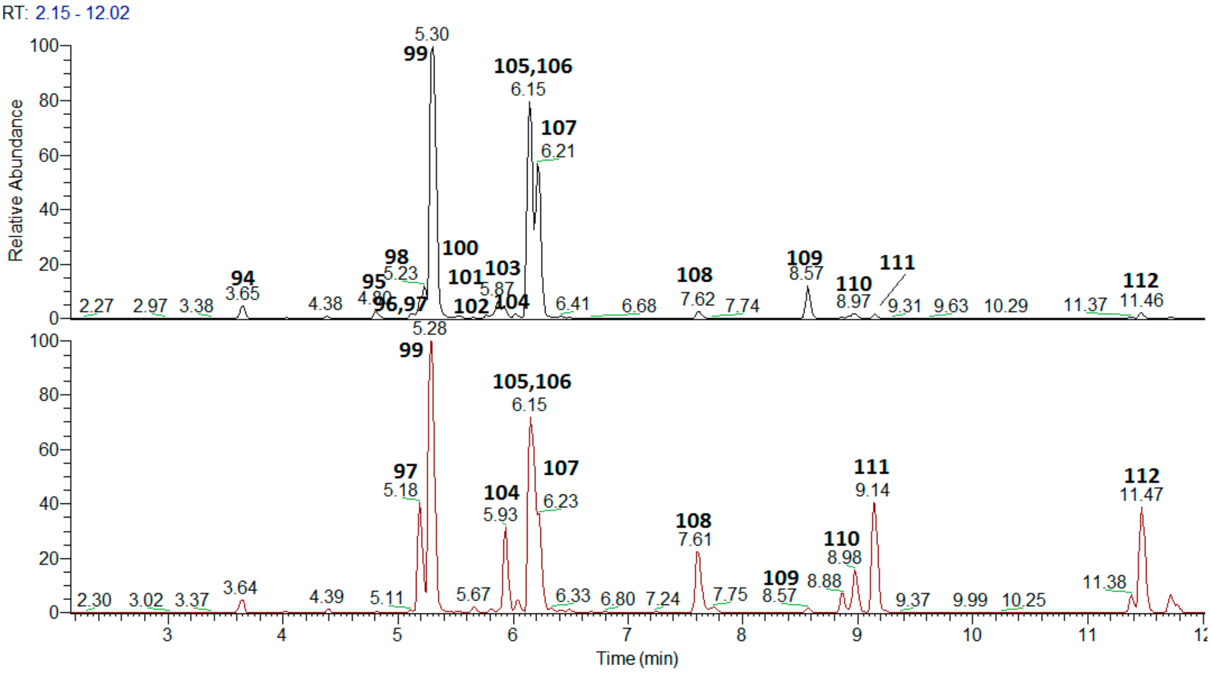

Figure. S2. EIC of flavonoids proceed with mass tolerance of 5 ppm as follows:
94 at *m/z* 595.1668 (595.1638-595.1698); **95** at *m/z* 447.0934 (447.0912-447.0956); **96** at *m/z* 595.1668 (595.1638-595.1698); **97** at *m/z* 463.0885 (463.0862-463.0908); **98** at *m/z* 593.1512 (593.1482-593.1542); **99** at *m/z* 463.0885 (463.0862-463.0908); **100** at *m/z* 623.1618 (623.1587-623.1649); **101** at *m/z* 579.1719 (579.1690-579.1748); **102** at *m/z* 577.1563 (577.1534-577.1592); **103** at *m/z* 447.0934 (447.0912-447.0956); **104** at *m/z* 461.1089 (461.1066-461.1112); **105** at *m/z* 607.1668 (607.1638-607.1698); **106** at *m/z* 477.1044 (477.1020-477.1068); **107** at *m/z* 609.1825 (609.1795-609.1855); **108** at *m/z* 301.0354 (301.0339-301.0369); **109** at *m/z* 271.0612 (271.0598-271.0626); **110** at *m/z* 299.0561 (299.0548-299.0576); **111** at *m/z* 593.1301 (593.1271-593.1331); **112** at *m/z* 739.1668 (739.1631-739.1705).

Plant material

Ostericum palustre/*Angelica pancicii* roots and aerial parts were collected near to Kumata hut, Vitosha Mts (42.594346ºN 23.250671ºE, altitude 1724 m a.s.l.), during the full flowering stage, in August, 2024.

The distribution of *O. palustre* is associated with places with abundant moisture and forest streams and swamps [1]. In the beech forests of Vitosha Mt. it is encountered only in the acidophilous forests and is registered in 20 sample plots totaling 3 ha, with 0.03 generative specimens per 1 m2 on the average. They are predominantly in the eastern divide of the mountain, where two largest locations are situated. In the other locations this species is represented by single specimens or by small groups, all situated in acidophilous beech coenoses along rivers or streams in the western and northern divide of Vitosha Mt. Only in the acidophilous beech forests there are favorable conditions for the development of *O. palustre* [2].

Vitosha Mt. is into the mountainous climatic region of the temperate continental climatic subregion of the European-continental climatic region. The territory of the mountain is characterized by relatively cold winters and hot summers for the Eastern European climate, with a characteristic spring-summer maximum of precipitation and a minimum in autumn. The mountainous variant of the temperate continental climate is characterized by low temperatures and high atmospheric humidity. The average annual air temperature fluctuates between 8°C to 0°C. The average January temperature varies from -1.7°C to -8.3°C, and the average July temperature from 21.2°C to 8°C. Above 1500 – 2000 m a.s.l., precipitations are in the range of 1000 – 1200 mm. The most days with precipitation are observed in the months of May – June (10 – 16 days),

and the least in the months of August – September (5 - 8 days). In the months of December, January and February, from 50 to 70% of the precipitation cases are from snow, 20 – 30% are from rain and about 10% are mixed [3].

The collected *O. palustre* inhabited one and the same plant community with the following taxa: *Senecio hercynicus* Herborg. (Asteraceae), *Epilobium angustifolium* L. (Onagraceae), *Cicerbita alpina* (L.) Wallr. (Asteraceae), *Filipendula ulmaria* (L.) Maxim. (Rosaceae).

References

1. Alexova D., Gyurova D. Vegetation and Flora of Vitosha Nature Park – Opportunities for Development of Botanical Tourism. *Proceeding of the Third Annual International Scientific Conference "ECOLOGISATION* 2011.
2. Gyurova, D.; Evstatieva, L. Distribution of Medicinal Plants in the Beech Forests of the Vitosha Nature Park. *PHYTOLOGIA BALCANICA* **2005**, *11*, 157–171.
3. Https://Pu-Vitosha.Com/Wp-Content/Uploads/2015/10/Sb.Abiotichni_f-Ri-APUV-28.10.2015.Pdf.
